# High-Stability Thick-Shell CdZnSeS/CdZnS/ZnS Green-Alloy Quantum Dots in Photoluminescent Diffuser-Plate Masterbatches

**DOI:** 10.3390/ma18235383

**Published:** 2025-11-28

**Authors:** Ziming Zhou, Dexia Zhou, Ning Li, Ya Liu, Zhaobing Tang, Siqi Jia, Xiao Wei Sun

**Affiliations:** 1 Institute of Advanced Displays and Imaging, Henan Academy of Sciences, Zhengzhou 450046, China; 2 Institute of Nanoscience and Applications, and Department of Electrical and Electronic Engineering, Southern University of Science and Technology, Shenzhen 518055, China

**Keywords:** CdZnSeS alloy quantum dots, orthogonal experiments, stability, diffuser plate masterbatches, ligand exchange

## Abstract

As a core component of emerging quantum-dot display technology, the stability of quantum-dot materials is crucial to determining the performance of quantum-dot photoluminescent diffuser plates. This study successfully synthesized high-stability thick-shell CdZnSeS/CdZnS/ZnS core–shell structured green-alloy quantum dots suitable for photoluminescent diffuser plates, providing an innovative solution for performance breakthroughs in this field. Through orthogonal experimental design, the synthesis parameters of the CdZnSeS alloy core were precisely optimized to achieve an ideal balance in emission wavelength, full width at half maximum (FWHM), and quantum yield (QY). Furthermore, by systematically adjusting ligands and synthesis parameters, a thick-shell CdZnSeS/CdZnS/ZnS core–shell structure was constructed, significantly improving the stability of the quantum dots. Critically, the replacement of the original oleic-acid ligands with tetradecylphosphonic-acid (TDPA) ligands at high temperature doubled the stability of the quantum-dot diffuser plates. Under extreme accelerated-aging conditions such as intense blue light, high temperature, and high humidity, the T90 lifetime of the diffuser plate exceeded 1000 h, and the xy chromaticity coordinate shift was strictly controlled within 1%, fully meeting the stringent commercial requirements. This achievement not only overcomes the stability bottleneck of quantum dots in the application of photoluminescent diffuser plates but also paves the way for their large-scale commercialization, promising to promote the development of display technology toward higher color gamut and longer lifetimes.

## 1. Introduction

Colloidal quantum-dot (QD) materials have shown great application potential in display, lighting, and biological imaging fields due to their narrow emission FWHM, high color purity, tunable emission wavelength, and excellent photoluminescent quantum yield [1,2,3]. In the display field, they can achieve precise color output across the entire visible spectrum by accurately controlling size and composition, meeting ultra-high color gamut standards. Technological breakthroughs and applications have been gradually realized in liquid-crystal display backlight modules, light-emitting diodes, and micro-LED color-conversion layers. Especially in the high-end TV and professional display markets, QD brightness-enhancement films and diffuser-plate technologies have become key directions for mainstream panel manufacturers to compete in layout, as they improve brightness uniformity and color-gamut coverage [4,5,6,7]. However, QD materials still face severe environmental stability challenges during actual device integration. Under high temperature, high humidity, and intense blue-light irradiation, they are prone to photothermal degradation, ligand desorption, and lattice oxidation, which seriously restrict their long-term service performance in consumer electronics, biomedicine, and other fields [4,8,9].

As a core component of current QD display technology, QD photoluminescent diffuser plates need to efficiently convert the excitation light emitted by blue LED chips in the backlight into pure green or red light and achieve uniform light emission from the screen through scattering structures. Their requirements for QD stability are much higher than those for solution or thin-film application scenarios. Under intense blue-light irradiation, photon energy is sufficient to induce the generation of surface-defect states and photo-corrosion of the core–shell structure in QDs. After continuous light irradiation, the photoluminescent quantum yield decreases significantly, accompanied by blue shift in the emission peak and broadening of the FWHM [10]; diffuser plates are often placed in closed backlight cavities, and the operating temperature accelerates ligand desorption and ion migration, destroying the carrier-confinement effect. Under hot and humid environments, the fluorescent lifetimes of QD films decay rapidly [11]; the packaging process cannot completely isolate water and oxygen permeation. High-humidity environments can induce hydrolysis and oxidation of shell sulfides. Although short-term water-molecule adsorption can passivate surface defects and temporarily increase fluorescence intensity, subsequent oxidation reactions will dominate, leading to irreversible fluorescence quenching [11,12,13]. Studies have shown that unprotected Cd-based QDs have short lifetimes and large chromaticity coordinate shifts under hot and humid conditions, failing to meet the basic requirements of consumer electronic products [4,8,9]; therefore, improving the quadruple stability (light, heat, water, and oxygen) of QDs under complex working conditions is not only a scientific problem at the material chemistry level but also an engineering bottleneck that determines whether they can be mass-produced and their application scenarios expanded [5,6,7].

Current problems of photoluminescent QD materials in terms of light, heat, water, and oxygen stability can be summarized into three types of mechanistic defects. First, lattice mismatch at the core–shell interface causes stress accumulation and defect proliferation. For example, the CdSe/ZnS system has a high lattice mismatch. Even with a gradient alloy transition layer, thick shells are prone to dislocations and microcracks, becoming non-radiative recombination centers, which significantly reduce the photoluminescent quantum yield and accelerate photothermal aging [14]; the lattice mismatch between CdTe and ZnS also leads to the proliferation of interface defects. The introduction of an intermediate shell can effectively alleviate this problem. For example, the CdTe/CdS/ZnS ternary structure can improve quantum yield and enhance stability [15,16,17]. Second, the instability of the dynamic balance of surface ligands leads to environmental sensitivity. Traditional weak coordination bonds of oleic acid or oleylamine ligands are easily dissociated in high-temperature or polar environments, exposing the QD surface and catalyzing oxidation reactions [18,19]; although thiol ligands can reduce surface defects through specific chemical bonds, they are prone to photocatalytic oxidation under light or oxidative environments, leading to fluorescence quenching. Long-chain oleic-acid ligands and siloxane ligands can inhibit photooxidation and block water and oxygen, respectively, and the mixed-ligand strategy has gradually become a new research direction [20,21]. Third, insufficient shell passivation causes exciton leakage and interface charge traps. Even with a multi-shell structure, if the shell has insufficient crystallinity or pores, photogenerated carriers may still tunnel to surface-defect states, undergoing Auger recombination or reacting with environmental molecules. Shell thickness is crucial for the passivation effect. Specific multi-shell structures can significantly improve light stability and water-oxygen barrier ability [9,17,22,23]. Experimental data show that most commercial green QDs exhibit obvious performance degradation in accelerated-aging tests. Intense blue-light irradiation can lead to irreversible lattice reconstruction, alkaline conditions accelerate the oxidation of thiol ligands, and acidic environments and temperature increases also exacerbate performance degradation. These factors together constitute the “stability bottleneck” in QD applications [4,5,9,20,24,25].

In recent years, the research community has proposed multi-level strategies to improve the environmental stability of QDs. In terms of shell-engineering optimization, thick shells or gradient alloy multi-shells are constructed to alleviate lattice stress. For example, the “well-type” structure achieves high solid-state photoluminescent quantum yield and breaks through the QLED lifetime bottleneck. Gradient alloy shells eliminate abrupt interfaces by continuously adjusting the component ratio, further improving stability [6,14,17,26,27]; in ligand chemistry innovation, mixed shells induced by oleylamine modification can significantly improve the hot and humid stability of QDs, and alkyl thiol ligands with stronger steric hindrance or antioxidants can improve the photostability of QD solutions [4,25,28]; in packaging barrier technology, low specific surface area AlO_x_ coatings can extend the lifetime of QDs [29], silica-based materials show excellent chemical inertness and optical transparency, and composite films constructed by atomic layer deposition (ALD) technology have low oxygen permeability [30,31]. Polymer-based composite coatings balance performance through molecular design. For example, phytic-acid-induced vesicle structures enhance water-vapor barrier ability, and organic/inorganic hybrid film packaging achieves an ultra-low water-vapor transmission rate [32,33]. Ultra-thin dielectric layers prepared by ALD also have unique advantages, and composite passivation structures can reduce leakage current and extend aging lifetime [34,35]; in terms of additive synergistic stability, the introduction of antioxidants and ultraviolet absorbers can quench free radicals and filter high-energy ultraviolet components, synergistically extending device lifetime [4,36]. In addition, although cadmium-free QD systems have lower toxicity and meet environmental regulation requirements, their intrinsic stability is poorer, requiring complex shell design. Although some structures achieve high photoluminescent quantum yield, their photostability is still inferior to that of their Cd-based counterparts, and they face problems of color-purity gap and cost. Their full-life cycle cost is expected to be comparable to that of Cd-based systems in the future [24,37,38,39,40]. Although these strategies have their own effects, they often need to sacrifice quantum yield, increase synthesis complexity, or raise costs, and a unified solution that balances performance, stability, and mass production feasibility has not yet been formed [1,3,7].

Despite the phased progress made in the above research, photoluminescent QD materials still have fundamental defects in the application of diffuser plates. Although the thick-shell strategy and ligand modification-induced mixed shells can improve thermal stability, excessively thick shells will increase the particle size of QDs, leading to sedimentation or agglomeration during coating, which destroys optical uniformity. The preparation process of mixed shells is complex and the batch consistency is poor, which is not conducive to mass production [4,6,9]; although packaging coatings are effective, they increase optical path loss and reduce overall light-extraction efficiency, and the sol–gel process has poor compatibility with roll-to-roll production [4,5]; the added amount of antioxidants is limited by solubility and compatibility, and excessive addition will lead to QD agglomeration or light scattering loss [4]. More importantly, existing studies mostly focus on a single stress source, lacking systematic optimization of the synergistic aging mechanism of light–heat–humidity. Under actual working conditions, local hot spots generated by blue-light irradiation will exponentially accelerate the water and oxygen permeation rate, while existing single-condition accelerated-aging tests fail to truly simulate this coupling effect, leading to a serious disconnect between laboratory data and on-site failure [4,9,10]. In recent years, various accelerated-aging test methods have been developed, such as temperature–humidity accelerated tests, “blue-light aging chamber” systems, and the Eyring equation to reveal the nonlinear synergistic effect of blue-light intensity and temperature on fluorescence quenching [35,41,42]. However, accelerated tests still have problems of material aging-rate differences and extrinsic failure, and the lifetime prediction error often exceeds expectations [43,44]. Feedback from the industry indicates that the current commercially available QD diffuser plates generally have short lifetimes, poor batch consistency, and insufficient yield in whole-machine tests. The fundamental reason is that the material-level stability has not broken through the “1000 h barrier”, which directly hinders the expansion of QD displays from high-end TVs to harsh-environment scenarios such as automotive and outdoor advertising.

To address the above industrial pain points, this study innovatively developed high-stability thick-shell CdZnSeS/CdZnS/ZnS core–shell structured green-alloy QDs suitable for photoluminescent diffuser plates. We selected CdZnSeS alloy QDs based on their unique advantages, including tunable emission within the visible-light range, narrow FWHM, minimal lattice-mismatch defects, high thermal stability, and mature synthesis technology. The research first precisely optimized the synthesis parameters of CdZnSeS alloy nucleation through orthogonal experiments, then systematically regulated the shell-growth kinetics to construct a dense ZnS shell with a thickness of >6 nm and critically used tetradecylphosphonic acid (TDPA) ligands to exchange the original oleic-acid ligands at 300 °C. As a result, the QD masterbatches achieved T90 lifetimes exceeding 1000 h under extreme accelerated-aging conditions of 100 mW/cm^2^ blue-light irradiation + 85 °C/85% RH, with chromaticity coordinate shifts Δx, Δy < 0.01, fully meeting the commercial requirements of display-panel whole machines. Compared with existing packaging technologies, the proposed scheme achieves longer lifetimes while maintaining high optical performance, providing a feasible path for the large-scale application of QD diffuser plates.

## 2. Materials and Methods

### 2.1. Chemicals

All chemicals used in this study were purchased directly without further treatment. The specific information of the drugs and reagents is as follows: cadmium acetate dihydrate (CdAH, 99.99%), zinc acetate dihydrate (ZnAH, 99.99%), zinc stearate (ZnSt, 98%), 1-octadecene (ODE, >90%), oleylamine (OAm, 80–90%), trioctylphosphine oxide (TOPO, 98%), polystyrene (PS), and isobornyl methacrylate (IBOMA) were purchased from Aladdin Scientific Corp., Shanghai, China; selenium powder (200 mesh, 99.998%), sulfur powder (100 mesh, 99.5%), oleic acid (OAc, 90%), and stearic acid (St) were purchased from Thermo Fisher Scientific Inc., Shanghai, China; zinc oxide (ZnO, 99%) was purchased from Sinopharm Chemical Reagent Co., Ltd., Shanghai, China; hexylphosphonic acid (HPA, 98%) and zinc oleate (Zn-OAc, 98%) were purchased from Shanghai Bidepharm Science&Technology Co., Ltd., Shanghai, China; tetradecylphosphonic acid (TDPA, 98%) and octadecylphosphonic acid (ODPA, 98%) were purchased from Guangdong Wengjiang Chemical Reagent Co., Ltd., Shaoguan, China; tributylphosphine (TBP, 98%) was purchased from Shanghai Macklin Biochemical Technology Co., Ltd., Shanghai, China; trioctylphosphine (TOP, 97%) was purchased from Strem Chemicals, Inc., Newburyport, MA, USA.

### 2.2. Orthogonal Experimental Design

#### 2.2.1. Optimization of CdZnSeS Alloy Core

To precisely optimize the synthesis parameters of the CdZnSeS alloy core, this study adopted the orthogonal experimental design method. Taking the photoluminescence (PL) wavelength, PL full width at half maximum (FWHM), PL quantum yield (QY), and reaction time required to achieve the optimal parameters of QDs as evaluation indicators, the influence laws of 5 key process parameters were explored, namely, Cd/Zn ratio, Se/S ratio, (Cd + Zn)/(Se + S) ratio, OAc/(Cd + Zn) ratio, and reaction temperature. Based on the results of preliminary pre-experiments, each parameter was set with 5 levels, and the L25(5^5^) orthogonal experimental scheme included 25 groups of experiments (recorded as Experiments A1–A25). The specific parameter levels and experimental design are shown in Table 1. Among them, the Cd/Zn ratio was set to 1, 0.2, 0.1, 0.04, and 0.02; the Se/S ratio was set to 10, 3, 1, 0.33, and 0.1; the (Cd + Zn)/(Se + S) ratio was set to 2, 1, 3, 0.5, and 0.33; the OAc/(Cd + Zn) ratio was set to 2.3, 2.6, 3, 4, and 6; and the reaction temperature was set to 280 °C, 300 °C, 320 °C, 260 °C, and 240 °C. Each group of experiments was repeated 3 times, and the average value was taken as the final result to ensure the reliability of the data.

#### 2.2.2. Optimization of ZnS Shell Coating Process

To precisely optimize the synthesis parameters of the ZnS shell-coating process, this study adopted the orthogonal experimental design method. Taking the PL wavelength, PL FWHM, PL QY of the final QDs, and the ratio of PL intensity after 144 h aging test to initial intensity as evaluation indicators, the influence laws of 3 key process parameters were explored, namely, shell-coating temperature, Zn source, and S source. Based on the results of preliminary pre-experiments, each parameter was set with 3 levels, and the L9(3^3^) orthogonal experimental scheme included 9 groups of experiments (recorded as Experiments B1–B9). The specific parameter levels and experimental design are shown in Table 2. Among them, the shell-coating temperature was set to 260 °C, 280 °C, and 300 °C; the Zn source was set to ZnAH, Zn-OAc, and ZnSt; and the S source was set to TBP-S, TOP-S, and DDT. Each group of experiments was repeated 3 times, and the average value was taken as the final result to ensure the reliability of the data.

### 2.3. Material Synthesis Process

#### 2.3.1. Preparation of Precursors

The 0.2 M Cd-OAc precursor was prepared as follows: 5.3325 g of CdAH, 15.07 g of OAc, and 65.5 g of ODE were added to a 500 mL round-bottom three-necked flask. Magnetic stirring was turned on, the temperature was raised to 160 °C and maintained for 60 min, and at least 6 vacuuming and nitrogen-filling cycles were performed to remove low-boiling solvents and water oxygen in the system until there were no solids or turbidity in the reaction. After cooling to 60 °C for storage, the 0.2 M Cd-OAc precursor was obtained.

The 2M TBP-Se precursor was prepared as follows: 1.58 g of Se powder and 10 mL of TBP were added to a 50 mL round-bottom three-necked flask. Magnetic stirring was turned on, the temperature was raised to 100 °C and maintained for 30 min, and at least 6 vacuuming and nitrogen-filling cycles were performed to remove low-boiling solvents and water oxygen in the system. After natural cooling to room temperature, the 2M TBP-Se precursor was obtained.

The 2M TBP-S precursor was prepared as follows: 0.64 g of S powder and 10 mL of TBP were added to a 50 mL round-bottom three-necked flask. Magnetic stirring was turned on, the temperature was raised to 100 °C and maintained for 30 min, and at least 6 vacuuming and nitrogen-filling cycles were performed to remove low-boiling solvents and water oxygen in the system. After natural cooling to room temperature, the 2M TBP-S precursor was obtained.

The S mixed solution was prepared as follows: 9 mL of 2M TBP-S precursor was thoroughly mixed with 28.40 g of ODE to obtain the S mixed solution.

#### 2.3.2. Synthesis of CdZnSeS Alloy Core

The synthesis of the CdZnSeS alloy core adopted the hot-injection method. Taking Experiment 1 of the orthogonal experiment as an example, the specific experimental steps are as follows: 2.2255 g of CdAH, 0.1833 g of ZnAH, 0.6116 g of ZnO, 10.85 g of OAc, and 53.51 g of ODE were added to a 500 mL four-necked round-bottom flask equipped with a high-temperature-resistant magnetic stir bar. Magnetic stirring was turned on, the temperature was raised to 150 °C and maintained for 30 min, and at least 6 vacuuming and nitrogen-filling cycles were performed to remove low-boiling solvents and water oxygen in the system. Subsequently, the temperature was raised to 280 °C and kept constant for 10 min; the pre-prepared mixed solution of 3.8 mL of 2M TBP-Se precursor and 0.38 mL of 2M TBP-S precursor was quickly injected into the reaction system, and timing was started simultaneously. During the reaction, samples were taken into toluene at regular intervals (1 min, 3 min, 10 min, 20 min, etc.) for subsequent PL and ultraviolet–visible absorption (Abs) spectrum characterization; after the reaction reached the present time, the reaction system was naturally cooled to terminate the reaction, and the CdZnSeS alloy-core QD solution was obtained.

#### 2.3.3. Synthesis of Thick-Shell Core–Shell Structured Quantum Dots

On the basis of the synthesis of the CdZnSeS core, the stepwise hot-injection method was used to construct the thick ZnS shell. Taking CdZnSeS/CdZnS/4ZnS as an example, the specific process is as follows: the CdZnSeS alloy core was synthesized according to the above experimental steps. At 10 min of reaction time, the temperature was raised to 300 °C and the pre-prepared mixture of 6 mL of 2M TBP-S precursor, 5 mL of 0.2M Cd-OAc precursor, and 21.3 g of ODE was uniformly added dropwise to the reaction system at a rate of 2.5 mL/min. After completion of dropping, the reaction was maintained at 300 °C for 25 min and then naturally cooled to terminate the reaction. After the reaction cooled to room temperature, 5.4219 g of ZnAH and 19.8 g of OAc were added to the flask, and the temperature was raised again. The temperature was maintained at 60 °C, 80 °C, 100 °C, 120 °C, 140 °C, and 160 °C for at least 30 min each, and at least 6 vacuuming and nitrogen-filling cycles were performed to remove low-boiling solvents and water oxygen in the system. Subsequently, the temperature was raised to 300 °C and the S mixed solution was uniformly added dropwise to the reaction system at a rate of 0.257 mL/min for 50 min; then, the reaction was maintained at 300 °C for 10 min; the S mixed solution was then added dropwise at a rate of 0.293 mL/min for 50 min, followed by maintaining the reaction at 300 °C for 10 min; the S mixed solution was added dropwise at a rate of 0.325 mL/min for 50 min, then the reaction was maintained at 300 °C for 10 min; finally, the S mixed solution was added dropwise at a rate of 0.36 mL/min for 50 min, and then, the reaction was maintained at 300 °C for 30 min; finally, it was naturally cooled to terminate the reaction, and the thick-shell CdZnSeS/CdZnS/4ZnS core–shell structured QD solution was obtained. Octane and ethanol were sequentially added to the reaction solution at a ratio of 1:1:2 for two rounds of QD cleaning, and finally, the QDs were dissolved in IBOMA at a concentration of 10 wt%.

#### 2.3.4. High-Temperature Ligand-Exchange Experiment

To improve the stability of QDs in diffuser plates, the high-temperature ligand-exchange method was used to replace some of the original oleic-acid ligands with different types of ligands to explore the experimental effect. The reasons for using high temperature for ligand exchange are, on the one hand, that high temperature is more likely to reach the desorption activation energy of the original ligands, accelerating the desorption of old ligands and the connection of new ligands, so that more ligands with strong binding abilities to QDs can be attached and, on the other hand, the hope that more ligands with stronger high-temperature resistance can be attached to QDs. Taking the experiment using TDPA as the exchange ligand as an example, the steps are as follows: after synthesizing the core–shell structured QDs according to the above experimental steps, 30 mL of QD stock solution and 23.67 g of ODE were added to a 250 mL four-necked round-bottom flask. Magnetic stirring was turned on, the temperature was raised to 150 °C and maintained for 30 min, and at least 6 vacuuming and nitrogen-filling cycles were performed to remove low-boiling solvents and water oxygen in the system. Subsequently, the temperature was raised to 300 °C, and the pre-prepared mixed solution of 0.5567 g of TDPA and 27.28 g of TOPO at 100 °C was quickly injected into the reaction system. Then, the reaction was maintained at 300 °C for 10 min, and then naturally cooled to terminate the reaction, obtaining TDPA-modified core–shell structured alloy QDs. Octane and ethanol were sequentially added to the reaction solution at a ratio of 1:1:2 for two rounds of QD cleaning, and finally the QDs were dissolved in IBOMA at a concentration of 10 wt%.

### 2.4. Preparation of Photoluminescent Masterbatches

Taking a representative experiment as an example, the specific steps for preparing photoluminescent masterbatches are as follows: the IBOMA solution of TDPA-modified core–shell structured alloy QDs was poured into 500 g of PS masterbatches at a concentration of 1 wt%, and the QDs were uniformly attached, coated, and embedded on the PS masterbatches by high-speed stirring. The PS masterbatches mixed with QDs were added to a high-temperature mixer and mixed at 180 °C for 30 min to obtain a molten mixture. Subsequently, the mixture was extruded and granulated in a twin-screw extruder at 200 °C to obtain photoluminescent QD masterbatches.

The core functions of the polymer matrix in QD diffuser plates include acting as a dispersion carrier, physical barrier, and enabling suitable processing. Common polymer matrices include polystyrene (PS), polymethyl methacrylate (PMMA), and polycarbonate (PC). The core reasons for selecting PS as the matrix in this work are its advantages in commercial applications, including low cost, light weight, high transparency, easy processability, and good compatibility with QD ligands.

However, the preparation scale of QD diffuser plates is large, requiring a huge amount of materials, and enterprises usually mix red and green QDs together for preparation, which is not convenient for the exploration of and improvement in the stability of green QDs in this paper. To facilitate the research and testing of the stability limit of green QD materials at the laboratory level, this study only conducted aging and testing of green QD photoluminescent masterbatches. In fact, since the diffuser plate has a three-layer structure, the middle QD light-emitting layer is wrapped between the two side diffuser-film layers, so its stability is usually better than that of masterbatches.

### 2.5. Performance Testing and Aging Evaluation Methods

#### 2.5.1. Basic Optical Performance Testing of Quantum Dots

The PL spectrum and Abs spectrum of QDs were tested with a fluorescence and absorption spectrometer (Duetta, Horiba, Ltd., Kyoto, Japan), where the excitation wavelength was set to 450 nm; the PL QY was measured at room temperature using a fluorescence quantum-yield testing system (FluoroMax, Horiba, Ltd., Kyoto, Japan); the particle size and morphology of QDs were observed by TEM (JEM-F200, JEOL Ltd., Tokyo, Japan). The samples were diluted with n-octane, dropped onto a copper mesh, and tested after vacuum drying.

#### 2.5.2. Accelerated-Aging Performance Evaluation

The accelerated-aging tests of PS masterbatches with attached QDs, QD masterbatches, and diffuser plates were carried out in a high-temperature, high-humidity blue-light aging chamber. The specific conditions were set as follows: blue-light source wavelength of 450 nm, light power density of 100 mW/cm^2^ (intense blue-light condition), aging temperature of 85 °C, and relative humidity of 85%RH (double-85); during aging, samples were taken at fixed intervals, cooled naturally, and tested. The brightness change and xy chromaticity coordinates of the diffuser plate were measured with a luminance colorimeter (BM-7A, TOPCON Corp., Tokyo, Japan); the T90 lifetime was defined as the time required for the brightness to decay to 90% of the initial brightness during aging, which was obtained by fitting the brightness–aging time curve; the chromaticity coordinate shift Δx, Δy was calculated according to the formulas Δx = xₜ − x_0_, Δy = yₜ − y_0_, where (x_0_, y_0_) is the initial chromaticity coordinate, and (xₜ, yₜ) is the chromaticity coordinate after aging for t time. All aging tests were repeated 3 times, and the average value was taken as the final result.

## 3. Results and Discussion

### 3.1. Orthogonal Optimization of CdZnSeS Alloy Core Synthesis Parameters

The composition and synthesis process of the CdZnSeS alloy core directly determine its optical performance. PL wavelength, PL FWHM, and PL QY are key indicators to measure whether QDs are suitable for photoluminescent diffuser plates—PL wavelength needs to be in the green band of 520–535 nm to meet display requirements, PL FWHM needs to be <30 nm to ensure high color purity, and PL QY needs to be >80% to ensure high luminous efficiency. Therefore, in this part, through orthogonal experimental design, the effects of Cd/Zn ratio, Se/S ratio, (Cd + Zn)/(Se + S) ratio, OAc/(Cd + Zn) ratio, and reaction temperature on core optical performance were systematically explored to achieve precise optimization of each parameter and obtain a CdZnSeS core with balanced optical performance.

According to the orthogonal experimental design in Section 2.2.1, a total of 25 groups of experiments were carried out (numbered Experiment A1–Experiment A25). Each group of experiments was performed according to the CdZnSeS core synthesis process in Section 2.3.2. During the reaction, samples were taken at 1 min, 3 min, 10 min, and 20 min (for some samples, since the spectrum was still changing and the reaction was not sufficient, samples were also taken at 30 min, 45 min, and 60 min). The PL spectrum and Abs spectrum of the samples at each time point were tested; the PL wavelength, PL FWHM, and PL QY were recorded; and the reaction time required for each group of experiments to achieve the optimal optical performance was determined. During the experiment, the amount of raw materials was precisely controlled according to the parameters of the orthogonal experimental scheme in Table 1. For example, the specific amount of Experiment A1 is described in Section 2.3.2. The amount of raw materials and reaction conditions of other experimental groups were adjusted according to the parameters in Table 1 to ensure a single variable and cover all level combinations. All experiments were carried out under nitrogen protection to avoid the interference of oxygen and moisture on the reaction. Each group of experiments was repeated three times, and the average value was used for subsequent analysis.

The PL and Abs spectrum test results of the cores of the 25 groups of orthogonal experiments are shown in Figures S1–S25. According to the results of this orthogonal experiment, the adjustable range of PL wavelength of the CdZnSeS alloy core is 528–630 nm, the variation range of PL FWHM is 28–40 nm, the variation range of PL QY is 1–71%, and the reaction time required to reach the optimal parameters is 8.8–30 min. The specific data results are shown in Table 1, which lists the time used for each group of experiments to achieve the narrowest PL FWHM and the highest PL QY, as well as the corresponding optical performance parameters.

Through the conventional analysis methods of orthogonal experiments, the mean response table and mean main-effect diagram analysis of each influencing factor on the optical performance parameters were performed for the time used to achieve the narrowest PL FWHM and the highest PL QY, and the corresponding eight optical performance parameters (see Figures S26–S33 for details). From the analysis results of each optical performance parameter corresponding to the sampling for the narrowest PL FWHM, the Cd/Zn ratio has the greatest influence on the PL wavelength. When the Cd/Zn ratio decreases, the PL wavelength shows an obvious blue shift trend. This is because the decrease in Cd content leads to an increase in the band gap of QDs, which in turn causes the blue shift in the PL wavelength, consistent with the quantum confinement effect theory [45]. The (Cd + Zn)/(Se + S) ratio (i.e., cation–anion ratio) is the most critical factor affecting PL FWHM and PL QY, indicating that it directly determines the crystallinity and surface-defect density of QDs [46]. When the (Cd + Zn)/(Se + S) ratio is 3, the ratio of cations to anions is appropriate, and there are fewer lattice defects during crystallization, so the PL FWHM is the smallest, and the PL QY is the highest. The Se/S ratio has the greatest influence on the reaction time, which may be due to the influence of the diffusion and rearrangement of Se and S elements in the alloy. When Se/S is 1, the reaction time required to achieve the narrowest PL FWHM is the longest; at this time, Se and S compete with each other for diffusion and rearrangement, which takes a long time. From the analysis results of each optical performance parameter corresponding to the sampling for the highest PL QY, the influencing factors of PL wavelength, PL FWHM, and PL QY are almost the same as those of the narrowest PL FWHM; only the influencing factors on the reaction time required to reach the highest PL QY are different. At 300 °C, the reaction kinetic rate is slow, which can not only ensure the sufficient nucleation and growth of QDs but also avoid the decomposition of ligands and the increase in surface defects caused by excessively high temperature [47]. The order of influence of each experimental factor on each optical performance parameter is summarized in Table 3.

In addition, the PL QY of some experimental groups is low (<10%), mainly because the parameter settings of the orthogonal experiment are too extreme, resulting in extremely poor nucleation conditions, the poor crystallinity of QDs, many surface defects, and a high probability of non-radiative recombination.

Through the design of this orthogonal experiment, the precise optimization of the synthesis parameters of the CdZnSeS alloy core was successfully achieved, and the process window with balanced optical performance was determined: Cd/Zn ratio of 0.04–0.10, Se/S ratio of 1–10, (Cd + Zn)/(Se + S) ratio of 2–3, OAc/(Cd + Zn) ratio of 4–6, and reaction temperature of about 300 °C. Within this parameter range, the PL wavelength of the CdZnSeS core can be adjusted to 547–594 nm, PL FWHM to <30 nm, and PL QY to >65%. Among them, the parameter combination of Experiment A18 (Cd/Zn = 0.04, Se/S = 1, (Cd + Zn)/(Se + S) = 2, OAc/(Cd + Zn) = 4, 300 °C) is the optimal core-synthesis parameter in this orthogonal experiment. Its PL wavelength of 547.7 nm is closer to the target green band (520–535 nm). In subsequent experiments, we further realized the blue shift in the PL wavelength by fine-tuning the Cd/Zn ratio to fully match the display requirements.

### 3.2. Construction of Thick-Shell CdZnSeS/CdZnS/ZnS Core–Shell Structure and Stability Improvement

Although the CdZnSeS alloy core has excellent optical performance, the exposed core surface has a large number of defect states, and it is prone to oxidation and ligand desorption under high-temperature and high-humidity environments, resulting in insufficient stability, which cannot meet the long-term service requirements of diffuser plates. The construction of the core–shell structure can reduce surface defects and block water and oxygen erosion through the physical wrapping and electronic passivation of the core by the shell, thereby improving the stability of QDs. Therefore, in this section, by systematically adjusting the shell-growth parameters, a thick-shell CdZnSeS/CdZnS/ZnS core–shell structure was constructed to explore the influence of the shell structure on the optical performance and stability of QDs and realize the synergistic improvement of optical performance and stability.

**Figure 1:**
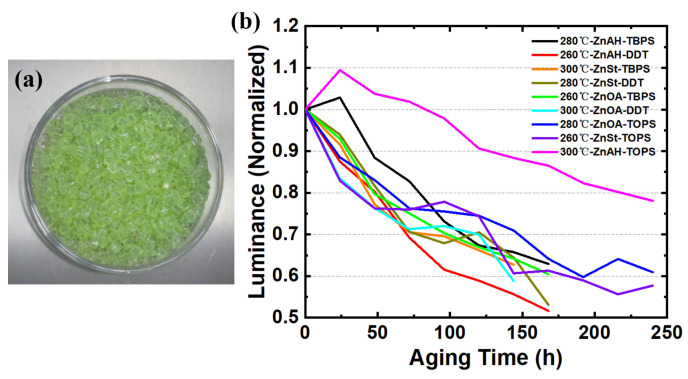
(**a**) PS masterbatches with attached QDs. (**b**) Brightness monitoring and change results of double-85 aging test for L9(3^3^) orthogonal experiment of ZnS shell-coating process optimization.

Considering the influence of lattice mismatch between CdZnSeS and ZnS shell, on the basis of the optimized CdZnSeS core synthesis in the previous section, a very thin CdZnS shell was coated as a transition layer. For the specific experimental steps, refer to Section 2.3.3. Then, the process of coating ZnS shell on CdZnSeS/CdZnS was explored and optimized. Similarly to the research method in Section 3.1, we also designed an orthogonal experiment. According to the orthogonal experimental design in Section 2.2.2, a total of nine groups of experiments were carried out (numbered Experiment B1–Experiment B9). Each group of experiments was performed according to the CdZnSeS shell-coating process in Section 2.3.3. Finally, the PL wavelength, PL FWHM, and PL QY of the QDs after shell coating, and the ratio of PL intensity after 144 h double-85 aging test to initial intensity were recorded. The 144 h double-85 aging test used PS masterbatches with attached QDs, without additives prepared, according to the method in Section 2.3.4 (Figure 1a). As an example, the specific amount of Experiment B9 is described in Section 2.3.3. The amount of raw materials and reaction conditions of other experimental groups were adjusted according to the parameters in Table 2. The molar amount was kept unchanged when changing different raw materials to ensure a single variable and cover all level combinations. All experiments were carried out under nitrogen protection to avoid the interference of oxygen and moisture on the reaction. Each group of experiments was repeated three times, and the average value was used for subsequent analysis.

According to the results of this orthogonal experiment, the PL wavelength after ZnS shell coating is basically stable between 539 and 544 nm, the fluctuation range of PL FWHM is 23–25 nm, the fluctuation range of PL QY is 78–90%, and the fluctuation range of the ratio of PL intensity after 144 h double-85 aging test to initial intensity is 55–89%. The specific data results are shown in Table 2, which lists the optical performance parameters of the QDs after shell coating in each group of experiments.

Through the conventional analysis methods of orthogonal experiments, the mean response table and mean main effect diagram analysis of each influencing factor on the optical performance parameters were performed for the four optical performance parameters of QDs (see Figures S34–S37 for details). From the analysis results of each optical performance parameter, probably because the same CdZnSeS/CdZnS QDs were used in the experiment, the PL wavelength and PL FWHM of the nine groups of experiments had little difference. It indicates that changing the reaction conditions during ZnS shell coating has little effect on these two parameters. As long as the ZnS layer grows, a PL wavelength blue shift of about 7 nm and a PL FWHM narrowing of about 4 nm can be achieved. The blue shift and narrowing are due to the diffusion and rearrangement of Zn and Se elements increasing the band gap of QDs and the uniformity of the size of the light-emitting region [48]. The most influential factor on PL QY is the S source. QDs coated with TOP-S have higher QY, which may be because TOP-S has lower activity and mild reactions, making the shell have better crystallinity and fewer defects during growth [49]. The most influential factor on the ratio of PL intensity after 144 h double-85 aging test to initial intensity (i.e., stability) is still the S source. Compared with TBP-S and DDT, QDs coated with TOP-S have significantly better stability, benefitting from the high boiling point and high stability of TOP ligands [50]. The order of influence of each experimental factor on each optical performance parameter is summarized in Table 4.

Through the design of this orthogonal experiment, the relatively optimal S source, Zn source, and reaction temperature for ZnS shell coating were successfully found to be TOP-S, ZnAH, and 300 °C, respectively. Among them, the condition combination of Experiment B9 is the optimal core synthesis parameter in this orthogonal experiment. It can also be clearly seen from the results of the double-85 aging test in Figure 1b that the QDs of Experiment B9 have higher stability.

**Figure 2:**
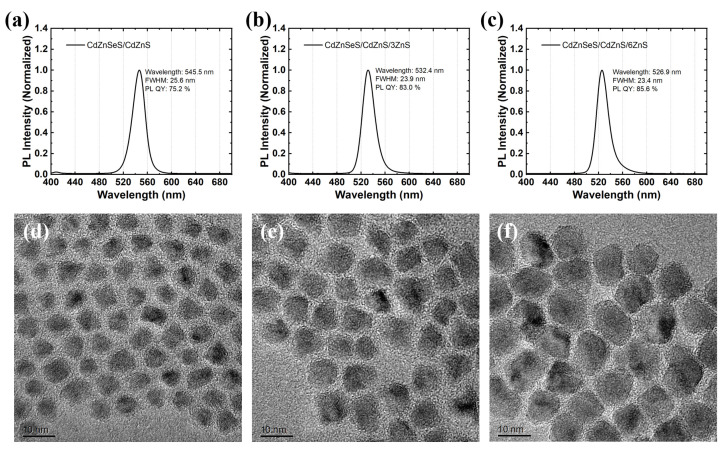
(**a**) CdZnSeS/CdZnS, (**b**) CdZnSeS/CdZnS/3ZnS, (**c**) PL spectra of CdZnSeS/CdZnS/6ZnS QDs. (**d**) CdZnSeS/CdZnS, (**e**) CdZnSeS/CdZnS/3ZnS, (**f**) transmission electron microscopy (TEM) images of CdZnSeS/CdZnS/6ZnS QDs.

Next, we further optimized the thickness of the ZnS shell and carried out the coating of three times and six times the amount of ZnS shell compared with the previous experiment to thicken the ZnS shell, thereby hoping to further improve the stability of QDs. We also tried a larger amount of ZnS shell coating. However, due to the excessively thick shell and large size, the solubility of QDs is poor and they cannot be well dispersed in IBOMA, so they are not suitable for subsequent aging tests and the application of diffuser plates. It can be seen from Figure 2a–c that the PL wavelengths of QDs without ZnS shell, with three times amount of ZnS shell, and with six times amount of ZnS shell are 545.5 nm, 532.4 nm, and 526.9 nm, respectively. With the increase in the shell, the wavelength further blue shifts due to element diffusion; the PL FWHMs are 25.6 nm, 23.9 nm, and 23.4 nm, respectively, which gradually narrow with the shell coating, indicating that the degree of alloying tends to be consistent and the band gap difference between QDs decreases [3,51]; the PL QYs are 75.2%, 83.0%, and 85.6%, respectively, indicating that with the increase in ZnS shell thickness, the surface defect states of the core are gradually covered, reducing the non-radiative recombination centers [52]. As shown in the TEM images of Figure 2d–f, with the gradual thickening of the ZnS shell, the size of QDs increases from 4.1–5.5 nm (the radius corresponding to this size is 2.05–2.75 nm, which is significantly smaller than the exciton Bohr radius of CdZnSeS (6–10 nm), indicating that these are indeed QD materials) to 6.8–8.0 nm and 10.0–11.4 nm, respectively.

**Figure 3:**
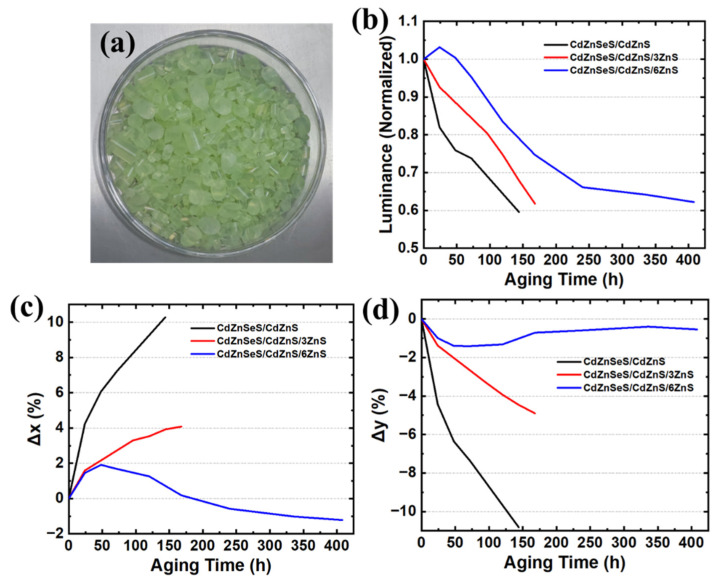
(**a**) QD masterbatches. Monitoring and change results of (**b**) brightness, (**c**) x chromaticity coordinate, and (**d**) y chromaticity coordinate of QD masterbatches under blue light + double-85 aging test.

Then, we prepared QD masterbatches of CdZnSeS/CdZnS, CdZnSeS/CdZnS/3ZnS, and CdZnSeS/CdZnS/6ZnS according to the method in Section 2.4 (Figure 3a) and carried out blue-light + double-85 aging tests on these three groups of QD masterbatches according to the aging-test method in Section 2.5. Figure 3b–d show the changes in brightness, x chromaticity coordinate, and y chromaticity coordinate of the three types of QDs during the entire aging process. From the changes in the three optical parameters, the QDs with thicker shells have slower brightness decay and smaller xy chromaticity coordinate drift, indicating that the physical barrier effect of the shell is enhanced, and water and oxygen are difficult to penetrate into the core, thus improving stability [53]. Using the same research method, we explored the influence of shell-coating time and precursor dropping rate on the stability of QDs. The specific aging-test analysis is shown in Figure S38a–c. When the injection rate is too fast and the reaction time is too short, Zn and S elements are rapidly deposited on the QD surface, easily forming an uneven shell (such as island growth) with relatively more surface pores, so the stability is relatively low [54]; however, if the injection rate is too slow and the reaction time is too long, the entire synthesis process will be too lengthy, which is not suitable for subsequent commercial applications. Finally, the optimized time for each ZnS shell coating amount is 60 min, and the precursor dropping time is 50 min. Through the optimization of shell thickness and shell-coating time, the brightness T90 lifetime of QD masterbatches under blue-light + double-85 accelerated-aging test can reach 329 h.

### 3.3. High-Temperature Ligand Exchange and Diffuser-Plate Stability Improvement

Although the thick-shell core–shell structure has significantly improved the intrinsic stability of QDs, the application of QDs in photoluminescent diffuser plates still faces complex working conditions of “solid-state dispersion–interface interaction–environmental erosion”: the high-temperature melting mixing and extrusion (190–220 °C) during the preparation of diffuser plates are likely to cause the desorption of the original oleic-acid ligands on the QD surface, and the high-temperature and high-humidity environment during service will further aggravate ligand dissociation, exposing QDs to the resin matrix, water, and oxygen, leading to failure behaviors such as agglomeration and oxidation [55]. Relevant studies have shown that the weak coordination bonds of traditional oleic-acid ligands (coordination between carboxyl groups and metal ions) are easily dissociated in high-temperature or polar environments, resulting in the exposure of QD surface defects and catalyzing oxidation reactions [56], which is one of the key bottlenecks restricting the stability of diffuser plates. Therefore, through the high-temperature ligand-exchange process, an attempt was made to replace some of the oleic-acid ligands with various other ligands, aiming to enhance the compatibility between QDs and PS matrix and inhibit ligand desorption and dissociation and hoping to achieve the improvement of diffuser-plate stability.

Starting from the optimal thick-shell core–shell structured QD synthesis scheme determined in the previous section, experiments were carried out according to the steps of the high-temperature ligand-exchange process in Section 2.3.4. At the same time, five control experiments were set up, original QDs without ligand exchange (Experiment A), QDs with ligand exchange using TDPA (Experiment B), ODPA (Experiment C), St (Experiment D), and OAm (Experiment E) at 300 °C, to explore the influence of ligand type on QD stability.

**Figure 4:**
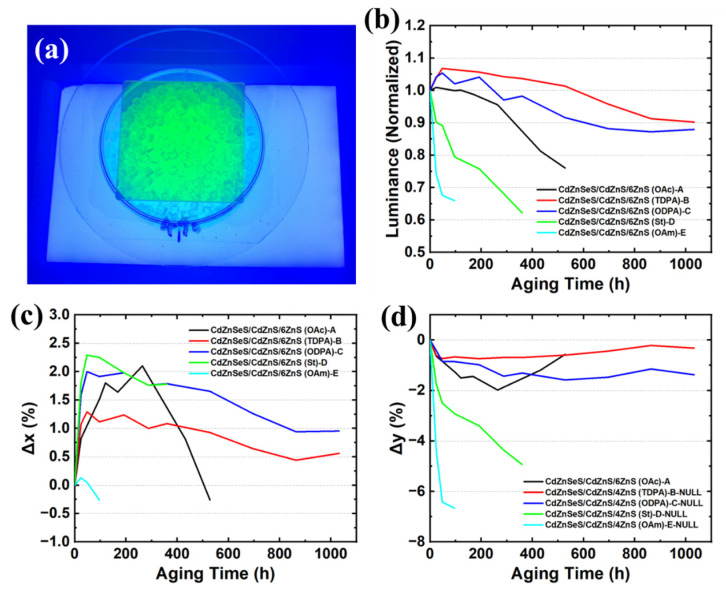
(**a**) QD masterbatches excited by blue backlight. Monitoring and change results of (**b**) brightness, (**c**) x chromaticity coordinate, and (**d**) y chromaticity coordinate of 5 control experiments under blue light + double-85 aging test.

Then, we prepared QD masterbatches of the five experiments according to the method in Section 2.4, and carried out blue light + double-85 aging tests on these five types of QD masterbatches according to the aging test method in Section 2.5. Figure 4a shows the QD masterbatches excited by blue backlight. The actual brightness of QDs can only be measured under blue-light excitation (the blue backlight has been filtered out with a filter during the test). Figure 4b–d show the changes in brightness, x chromaticity coordinate, and y chromaticity coordinate of the five types of QDs during the entire aging process. From the changes in the three optical parameters, phosphonic acid ligands, such as TDPA and ODPA, can significantly improve the stability of QDs, reduce brightness decay and color drift during aging. This should be attributed to the stronger coordination ability and higher boiling point of phosphonic-acid ligands, which make QDs not easy to desorb during high-temperature mixing and extrusion (200 °C) and long-term aging (85 °C), continuously covering the QD surface, avoiding the exposure of defect states, and thus reducing brightness decay caused by non-radiative recombination [57]. However, ODPA with a longer chain is not as effective as TDPA in improving stability, which should be because ODPA has a longer chain length and larger steric hindrance, and the amount that can be exchanged onto QDs is less than that of TDPA with a shorter chain, resulting in less improvement in QD performance [58]. In contrast, OAm and St make the stability of QDs worse, on the one hand, because these two ligands are not superior to OAc in terms of boiling point and binding energy and, on the other hand, as ligand exchange causes more voids and defects in the QDs themselves [59]; the combination of these two reasons makes the QD performance not improve but decrease.

Fourier transform infrared spectroscopy (FTIR) measurements were performed on QDs before and after ligand exchange, confirming that TDPA successfully replaced the OAc through ligand exchange. Emission and excitation spectra of pure QDs and QDs in PS were measured, showing almost no changes before and after the addition of PS. This indicates that the PS matrix can well preserve the intrinsic luminescent properties of QDs without affecting their excitation and emission. For detailed experimental results, refer to Figures S39 and S40 in the Supplementary Materials.

Through the high-temperature ligand exchange of TDPA, the prepared QD masterbatches achieved a brightness T90 lifetime of 1032 h under blue-light + double-85 accelerated-aging test. At the same time, Δx and Δy are only 0.85% and 0.52%, respectively, which fully meet the commercial requirements of diffuser plates.

## 4. Conclusions

To solve the core bottleneck of insufficient light, heat, water, and oxygen stability faced by QDs in the application of photoluminescent diffuser plates, this study systematically carried out the synthesis and performance optimization research of high-stability thick-shell CdZnSeS/CdZnS/ZnS core–shell structured green-alloy QDs. Through the L25(5^5^) orthogonal experiment, the synthesis parameters of the CdZnSeS alloy core were precisely optimized, and the optimal process window was determined: Cd/Zn ratio of 0.04–0.10, Se/S ratio of 1–10, (Cd + Zn)/(Se + S) ratio of 2–3, OAc/(Cd + Zn) ratio of 4–6, and reaction temperature of 300 °C, realizing the balance of optical performance with PL wavelength of 547–594 nm, FWHM <30 nm, and quantum yield >65%. On this basis, the CdZnSeS/CdZnS/ZnS thick-shell core–shell structure was constructed by the stepwise hot-injection method. Through the L9(3^3^) orthogonal experiment, TOP-S was determined as the optimal S source. Combined with the optimization of shell thickness (six times the amount of ZnS), the accelerated-aging T90 lifetime of QD masterbatches increased to 329 h. Furthermore, the replacement of the original oleic-acid ligands with tetradecylphosphonic acid (TDPA) ligands at 300 °C significantly enhanced the stability of QDs. The PS matrix selected in this study not only serves as a dispersion carrier for QDs but also its physical barrier effect synergizes with the thick-shell structure and TDPA ligand modification, collectively ensuring the long-term stability of the diffuser plate under extreme conditions. Meanwhile, the low cost and process compatibility of PS provide feasibility for the large-scale commercial application of QD diffuser plates. Finally, under the extreme accelerated-aging conditions of 100 mW/cm^2^ intense blue light + 85 °C/85% RH, the T90 lifetime of QD masterbatches exceeded 1000 h, and the xy chromaticity coordinate shift was <1%, fully meeting the commercial requirements.

Through the synergistic strategy of core parameter optimization, thick-shell construction, and ligand modification, this study effectively overcomes the stability problem of QD diffuser plates, provides a feasible scheme for their large-scale commercial application, and is of great significance for promoting the development of display technology toward high color gamut and long lifetime. Admittedly, this paper is only based on laboratory-level testing and research. There will be more and more complex problems and challenges in the actual engineering-application stage. This paper only provides a referenceable optimization path for the commercial application development of QDs in diffuser plates.

## Figures and Tables

**Table 1:** L25(5^5^) orthogonal experimental scheme design parameters and representative optical performance parameter results for CdZnSeS alloy-core optimization.

No.	Cd/Zn Ratio	Se/S Ratio	(Cd + Zn)/(Se + S) Ratio	OAc/(Cd + Zn) Ratio	Temperature (°C)	Most Narrow PL FWHM				Highest PL QY			
						Time (min)	PL Wavelength (nm)	PL FWHM (nm)	PL QY (%)	Time (min)	PL Wavelength (nm)	PL FWHM (nm)	PLQY (%)
A1	1	10	2	2.3	280	10	597.5	54.9	2.6	10	597.5	54.9	2.6
A2	1	3	1	2.6	300	30	631.9	41.7	17.5	45	635.5	44.5	18.1
A3	1	1	3	3	320	30	614.6	35.1	14.6	3	605.2	41.5	22.8
A4	1	0.33	0.5	4	260	10	615.3	32.1	2.4	30	619.9	40.5	4.0
A5	1	0.1	0.33	6	240	20	629.9	35.9	1.1	1	591.1	69.5	1.4
A6	0.2	10	1	3	260	3	609.3	30.3	3.7	20	608.2	48.7	23.3
A7	0.2	3	3	4	240	30	577.6	32.7	8.2	45	578.6	35.1	10.4
A8	0.2	1	0.5	6	280	60	600.9	60.3	15.1	10	601.9	70.7	16.4
A9	0.2	0.33	0.33	2.3	300	3	584.7	47.0	7.6	10	584.7	70.6	24.4
A10	0.2	0.1	2	2.6	320	3	585.1	38.0	52.0	3	585.1	38.0	52.0
A11	0.1	10	3	6	300	20	594.2	27.9	66.7	30	595.5	30.7	67.6
A12	0.1	3	0.5	2.3	320	10	581.4	47.9	33.4	3	590.1	48.0	36.2
A13	0.1	1	0.33	2.6	260	20	605.2	64.5	4.1	10	605.2	65.1	6.1
A14	0.1	0.33	2	3	240	30	584.1	30.6	9.0	45	589.1	31.5	9.8
A15	0.1	0.1	1	4	280	10	570.5	47.0	27.6	10	570.5	47.0	27.6
A16	0.04	10	0.5	2.6	240	10	599.2	52.7	4.6	10	599.2	52.7	4.6
A17	0.04	3	0.33	3	280	10	601.2	63.5	4.8	3	597.9	63.7	5.9
A18	0.04	1	2	4	300	20	547.7	29.4	68.0	60	547.7	31.2	69.3
A19	0.04	0.33	1	6	320	20	529.1	31.0	38.6	1	551.1	31.3	47.2
A20	0.04	0.1	3	2.3	260	20	590.1	30.9	63.6	20	590.1	30.9	63.6
A21	0.02	10	0.33	4	320	3	586.8	59.3	0.0	1	580.7	40.6	13.9
A22	0.02	3	2	6	260	10	567.1	44.6	17.5	3	580.7	30.9	41.4
A23	0.02	1	1	2.3	240	20	586.1	58.7	24.3	20	586.1	58.7	24.3
A24	0.02	0.33	3	2.6	280	10	575.9	29.5	54.6	10	575.9	29.5	54.6
A25	0.02	0.1	0.5	3	300	1	578.6	53.1	25.3	1	578.6	53.1	25.3

**Table 2:** L9(3^3^) orthogonal experimental scheme design parameters and representative optical performance parameter results for ZnS shell-coating process optimization.

No.	Temperature (°C)	Zn Source	S Source	PL Wavelength (nm)	PL FWHM (nm)	PL QY (%)	Retention Ratio of PL Intensity After 144 h Aging Test (%)
B1	280	ZnAH	TBP-S	540.72	23.51	81.5	65.75
B2	260	ZnAH	DDT	543.46	23.78	84	55.62
B3	300	ZnSt	TBP-S	539.34	23.35	85.7	62.75
B4	280	ZnSt	DDT	540.72	23.57	89.3	64.46
B5	260	ZnOA	TBP-S	540.72	23.63	85.3	64.18
B6	300	ZnOA	DDT	542.78	23.88	89.8	58.81
B7	280	ZnOA	TOP-S	541.4	24.17	80.4	70.94
B8	260	ZnSt	TOP-S	541.4	23.48	78.6	60.66
B9	300	ZnAH	TOP-S	540.37	23.51	79.4	88.37

**Table 3:** Order of influence of each experimental factor on each optical performance parameter analyzed from L25(5^5^) orthogonal experiment results for CdZnSeS alloy-core optimization.

Importance Ranking (1: Most, 5: Least)		1	2	3	4	5
**Most Narrow PL FWHM**	**Reaction Time**	Se/S Ratio	OAc/(Cd + Zn) Ratio	Cd/Zn Ratio	(Cd + Zn)/(Se + S) Ratio	Temperature
	**PL Wavelength**	Cd/Zn Ratio	(Cd + Zn)/(Se + S) Ratio	OAc/(Cd + Zn) Ratio	Se/S Ratio	Temperature
	**PL FWHM**	(Cd + Zn)/(Se + S) Ratio	Se/S Ratio	Temperature	Cd/Zn Ratio	OAc/(Cd + Zn) Ratio
	**PL QY**	(Cd + Zn)/(Se + S) Ratio	Cd/Zn Ratio	Temperature	Se/S Ratio	OAc/(Cd + Zn) Ratio
**Highest PL QY**	**Reaction Time**	Temperature	OAc/(Cd + Zn) Ratio	(Cd + Zn)/(Se + S) Ratio	Se/S Ratio	Cd/Zn Ratio
	**PL Wavelength**	Cd/Zn Ratio	OAc/(Cd + Zn) Ratio	Temperature	(Cd + Zn)/(Se + S) Ratio	Se/S Ratio
	**PL FWHM**	(Cd + Zn)/(Se + S) Ratio	OAc/(Cd + Zn) Ratio	Temperature	Se/S Ratio	Cd/Zn Ratio
	**PL QY**	(Cd + Zn)/(Se + S) Ratio	Temperature	Cd/Zn Ratio	OAc/(Cd + Zn) Ratio	Se/S Ratio

**Table 4:** Order of influence of each experimental factor on each optical performance parameter analyzed from L9(3^3^) orthogonal experiment results for ZnS shell-coating process optimization.

Importance Ranking (1: Most, 3: Least)	1	2	3
PL Wavelength	S Source	Zn Source	Temperature
PL FWHM	Zn Source	S Source	Temperature
PL QY	S Source	Zn Source	Temperature
Luminance Stability	S Source	Temperature	Zn Source

## Data Availability

The original contributions presented in this study are included in the article/Supplementary Materials. Further inquiries can be directed to the corresponding authors.

## Supplementary Materials

The following supporting information can be downloaded at https://www.mdpi.com/article/10.3390/ma18235383/s1, Figures S1–S25: Abs and PL Spectra of Experiment A1–A25 samples; Figures S26–S33: Mean response table and mean main effect diagram analysis of each influencing factor on the optical performance parameters were performed for the time used to achieve the narrowest PL FWHM and the highest PL QY, and the corresponding eight optical performance parameters; Figures S34–S37: mean response table and mean main effect diagram analysis of each influencing factor on the optical performance parameters were performed for the four optical performance parameters of QDs; Figure S38: Monitoring and change results of (a) brightness, (b) x chromaticity coordinate, (c) y chromaticity coordinate of QD masterbatches under blue-light + double-85 aging test; Figure S39: Fourier transform infrared spectroscopy (FTIR) transmission spectra of QDs before (black line) and after (red line) ligand exchange; Figure S40: (a) Emission spectrum of pure QDs; (b) Emission spectrum of QDs in PS; (c) Excitation spectrum of pure QDs; (d) Excitation spectrum of QDs in PS.
