# Supplementary material for: High-Stability Thick-Shell CdZnSeS/CdZnS/ZnS Green-Alloy Quantum Dots in Photoluminescent Diffuser-Plate Masterbatches
Source: Materials (Basel). 2025 Nov 28;18(23):5383. doi: 10.3390/ma18235383 (PMC12693124; doi:10.3390/ma18235383)
Supplement: Supplementary file 1 [file materials-18-05383-s001.zip › materials-3990807-supplementary.pdf]

Supporting Information

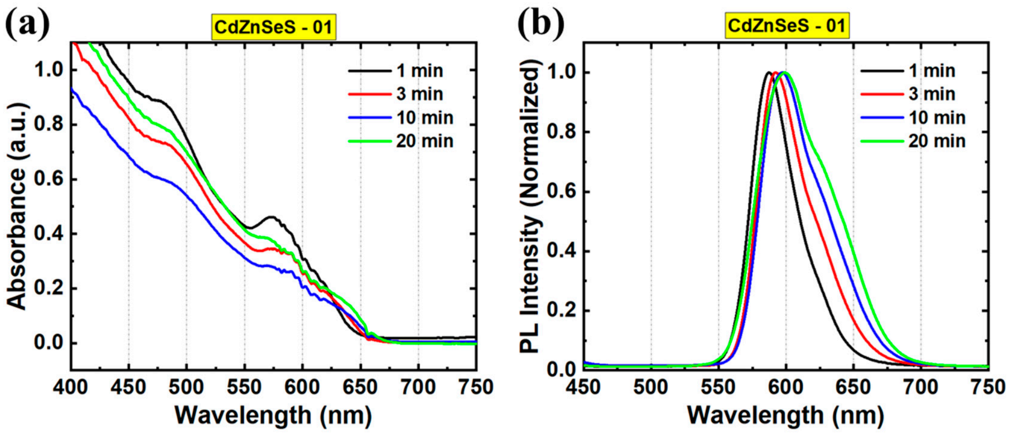

Figure S1. (**a**) Abs and (**b**) PL Spectra of Experiment A1 samples.

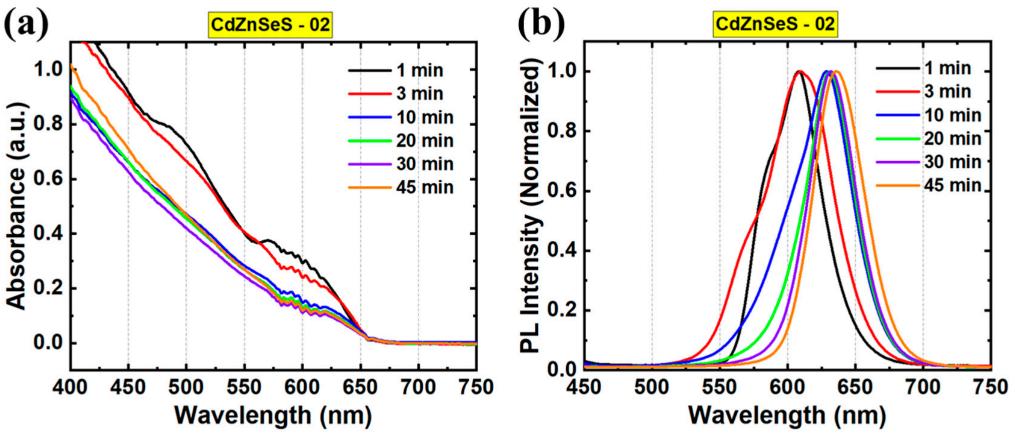

Figure S2. (**a**) Abs and (**b**) PL Spectra of Experiment A2 samples.

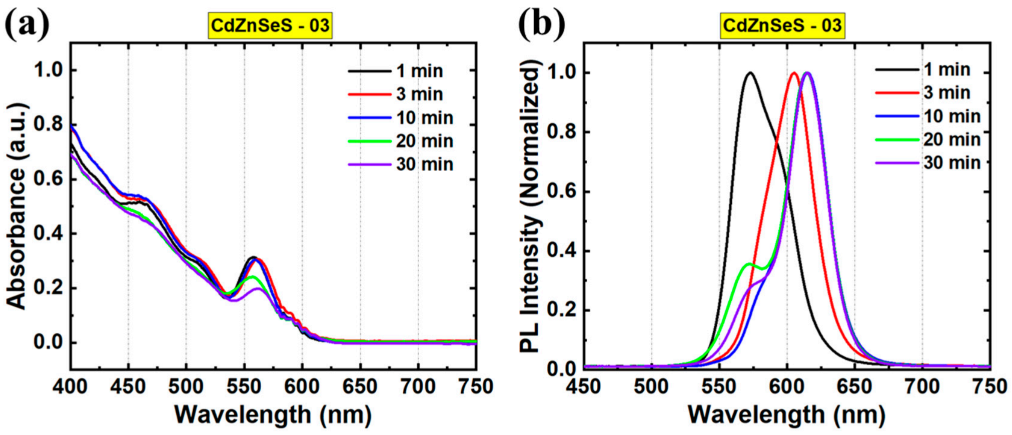

Figure S3. (**a**) Abs and (**b**) PL Spectra of Experiment A3 samples.

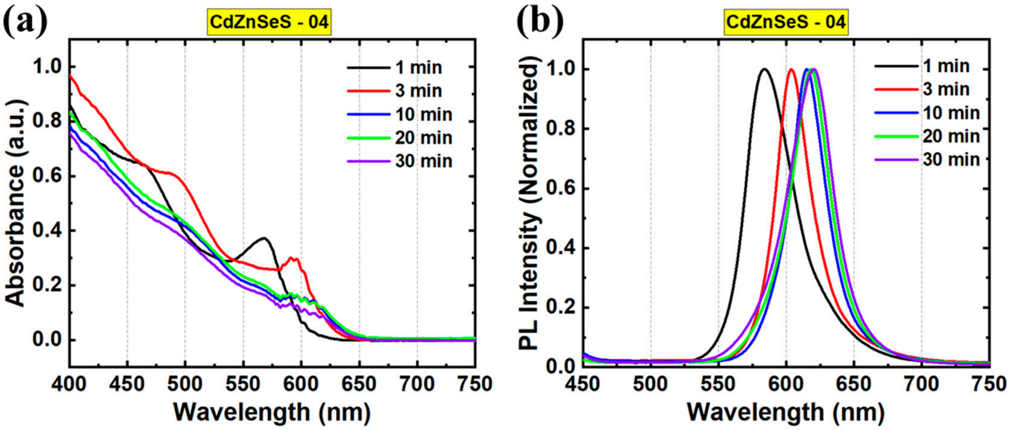

Figure S4. (**a**) Abs and (**b**) PL Spectra of Experiment A4 samples.

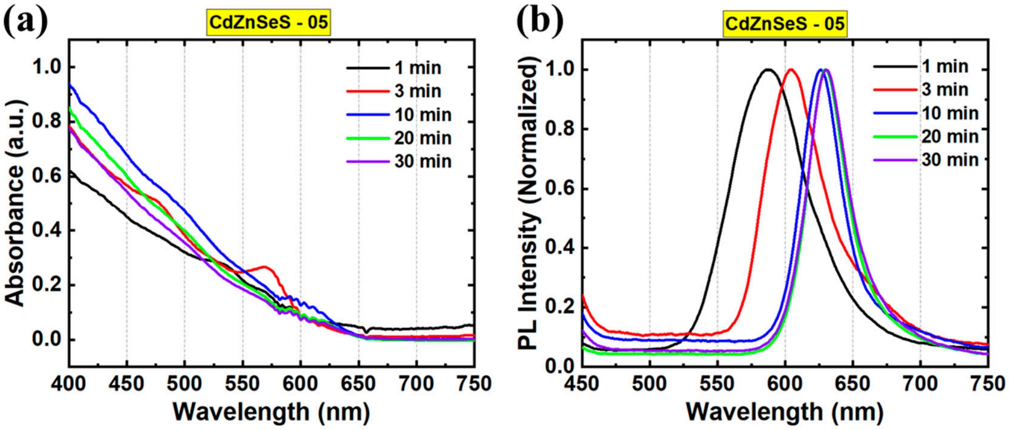

Figure S5. (**a**) Abs and (**b**) PL Spectra of Experiment A5 samples.

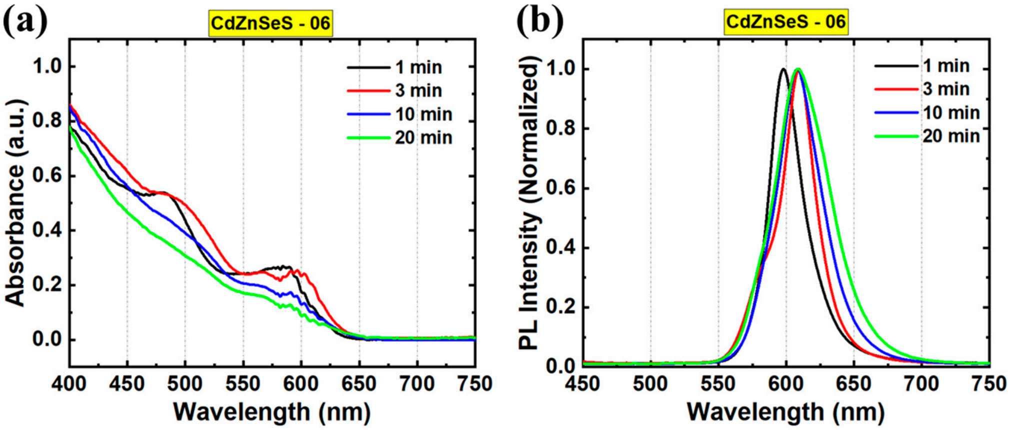

Figure S6. (**a**) Abs and (**b**) PL Spectra of Experiment A6 samples.

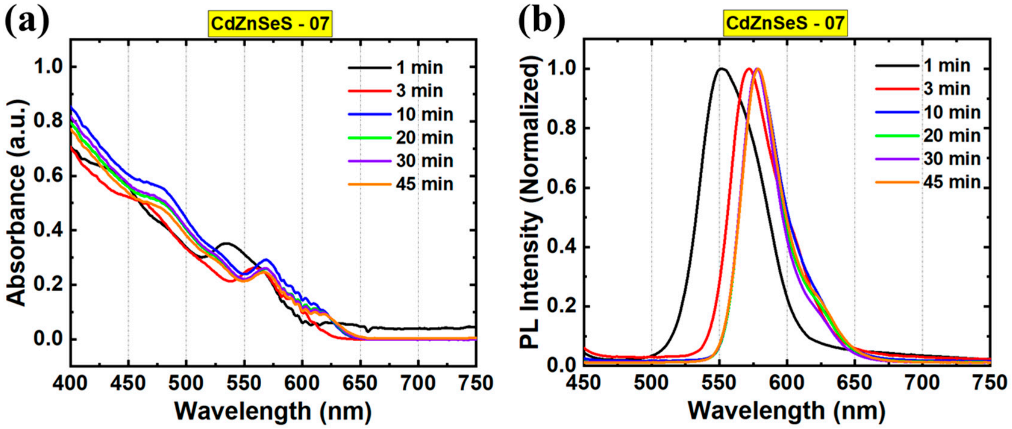

Figure S7. (**a**) Abs and (**b**) PL Spectra of Experiment A7 samples.

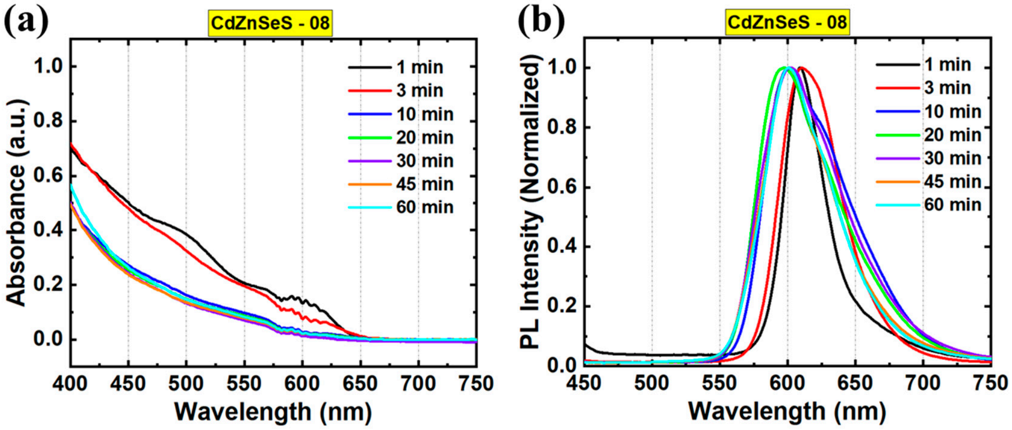

Figure S8. (**a**) Abs and (**b**) PL Spectra of Experiment A8 samples.

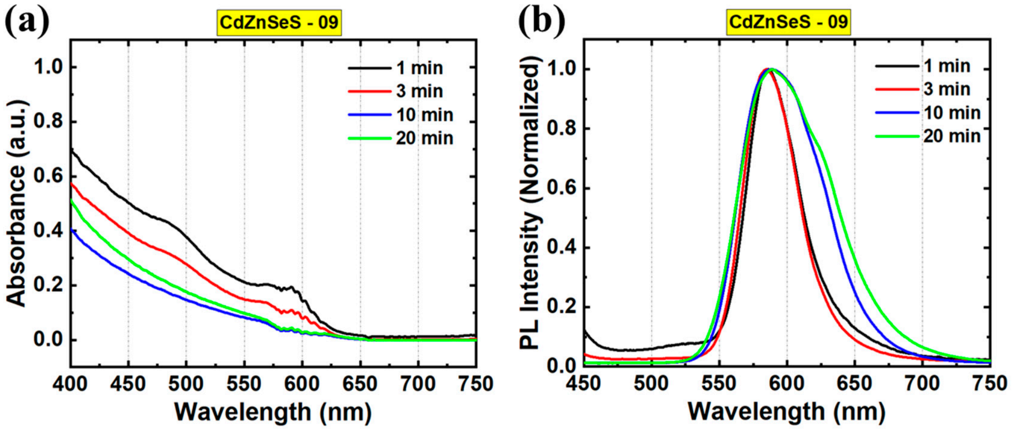

Figure S9. (**a**) Abs and (**b**) PL Spectra of Experiment A9 samples.

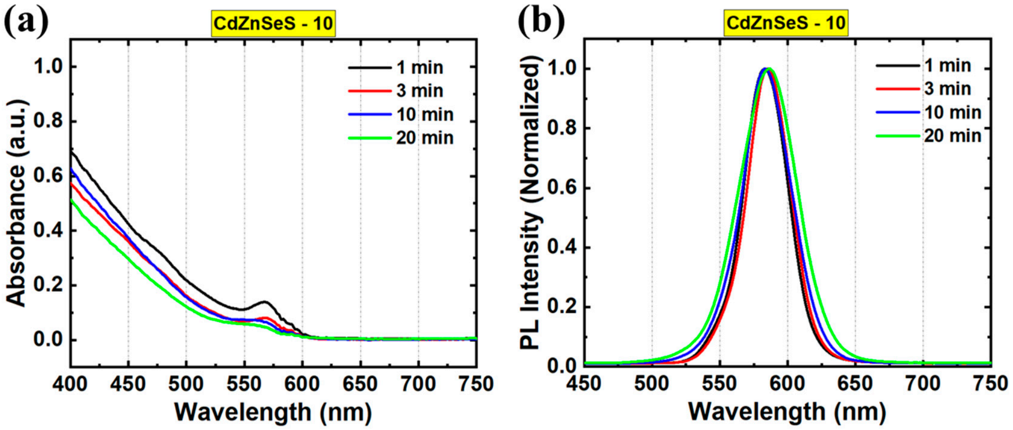

Figure S10. (**a**) Abs and (**b**) PL Spectra of Experiment A10 samples.

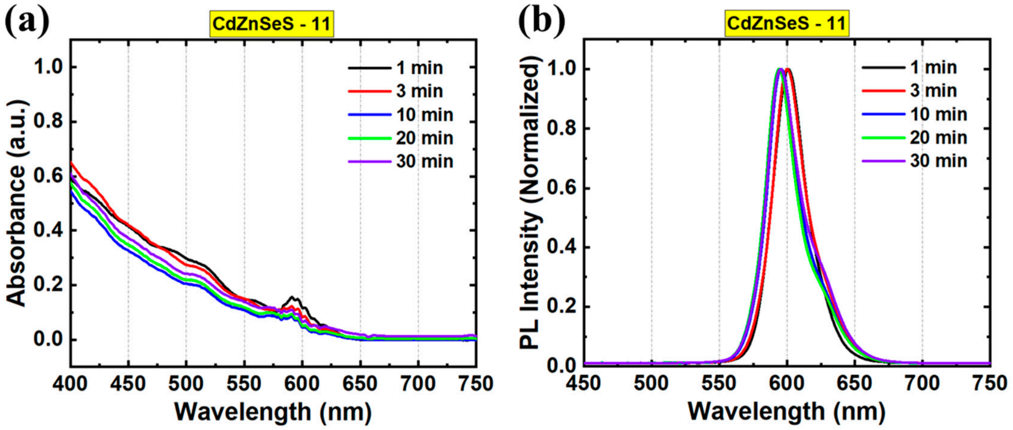

Figure S11. (**a**) Abs and (**b**) PL Spectra of Experiment A11 samples.

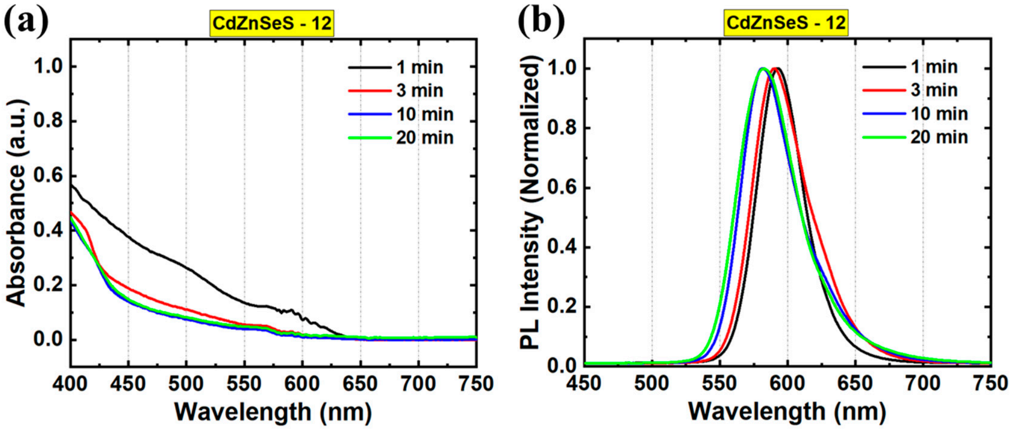

Figure S12. (**a**) Abs and (**b**) PL Spectra of Experiment A12 samples.

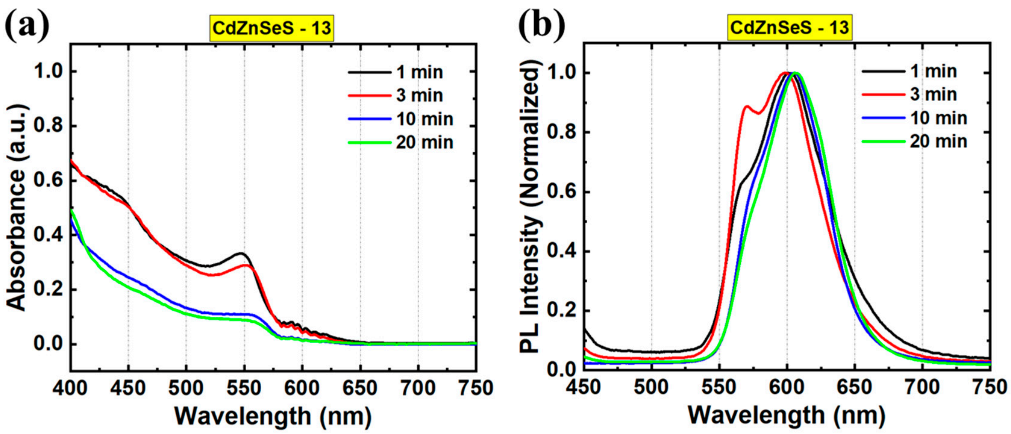

Figure S13. (**a**) Abs and (**b**) PL Spectra of Experiment A13 samples.

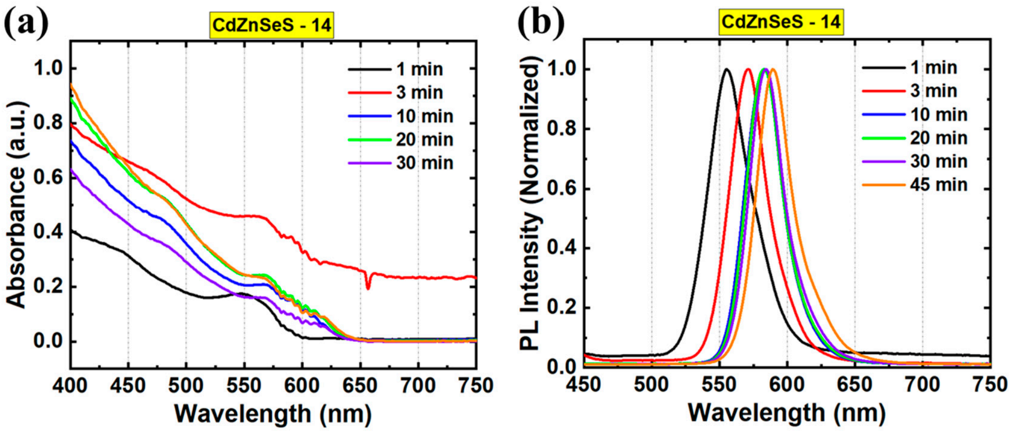

Figure S14. (**a**) Abs and (**b**) PL Spectra of Experiment A14 samples.

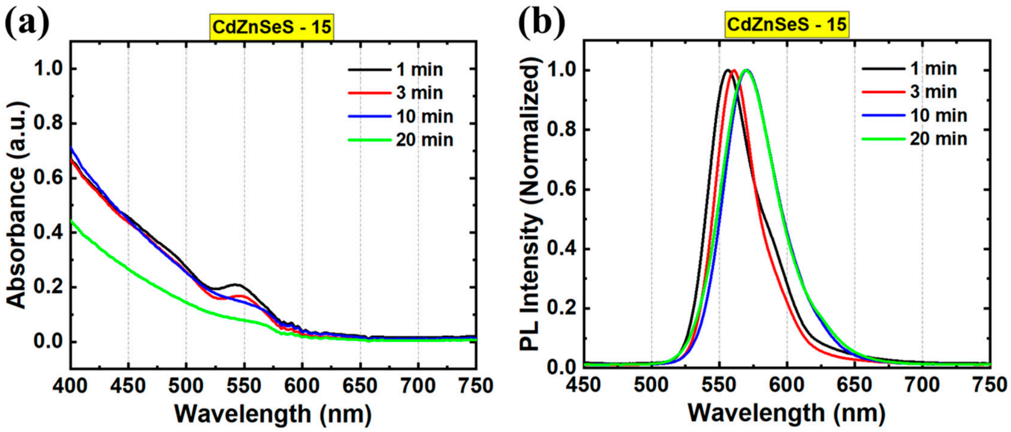

Figure S15. (**a**) Abs and (**b**) PL Spectra of Experiment A15 samples.

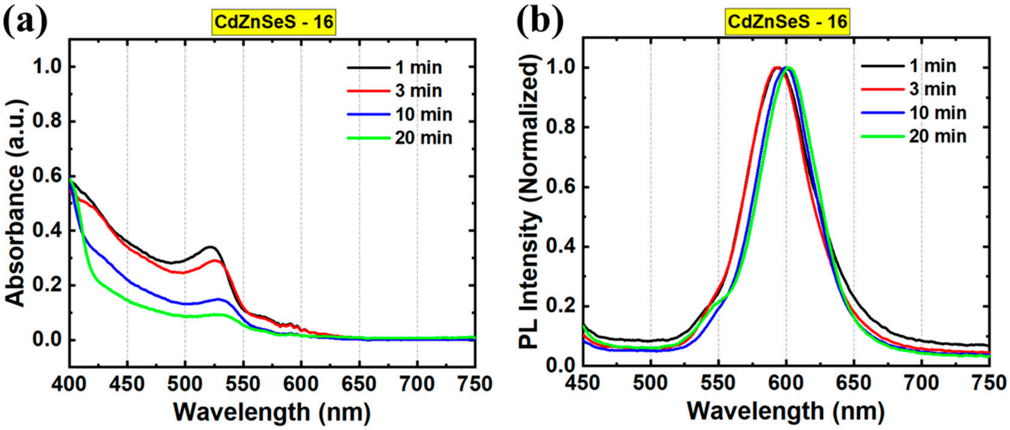

Figure S16. (**a**) Abs and (**b**) PL Spectra of Experiment A16 samples.

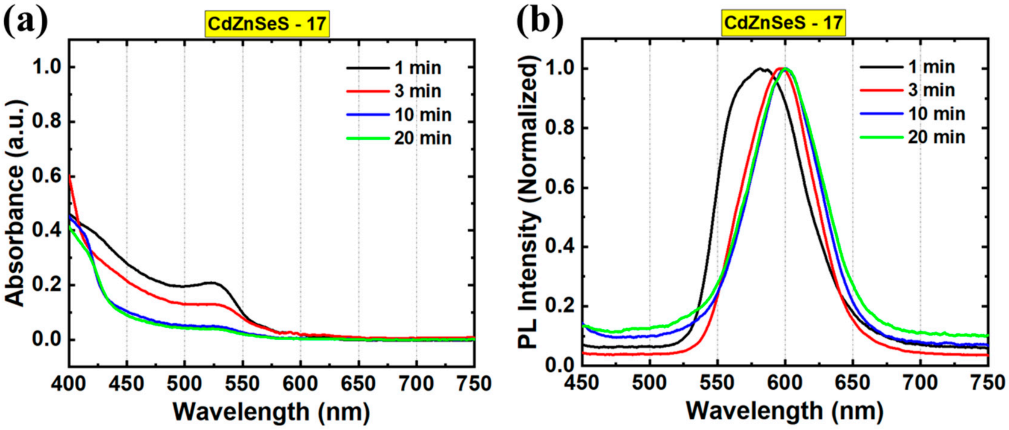

Figure S17. (**a**) Abs and (**b**) PL Spectra of Experiment A17 samples.

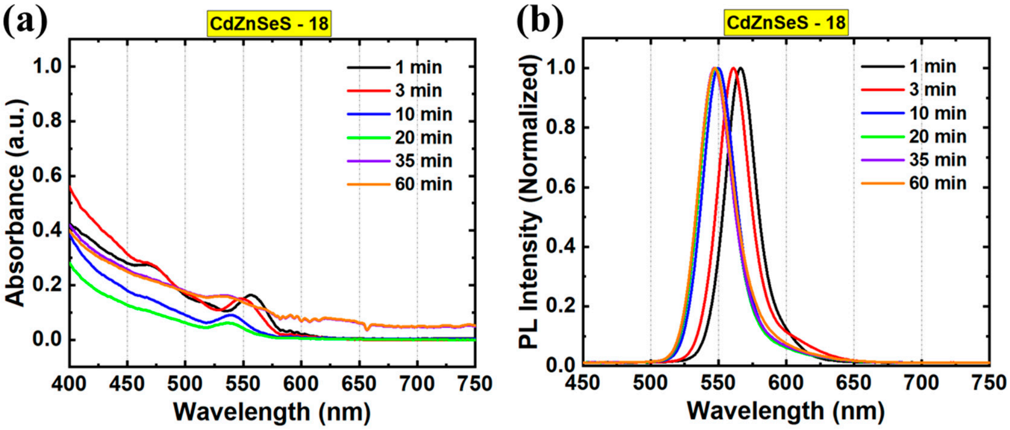

Figure S18. (**a**) Abs and (**b**) PL Spectra of Experiment A18 samples.

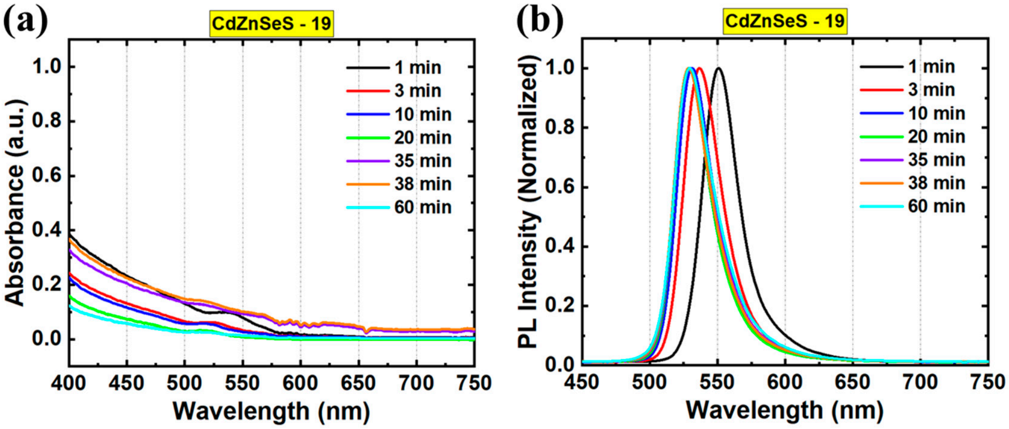

Figure S19. (**a**) Abs and (**b**) PL Spectra of Experiment A19 samples.

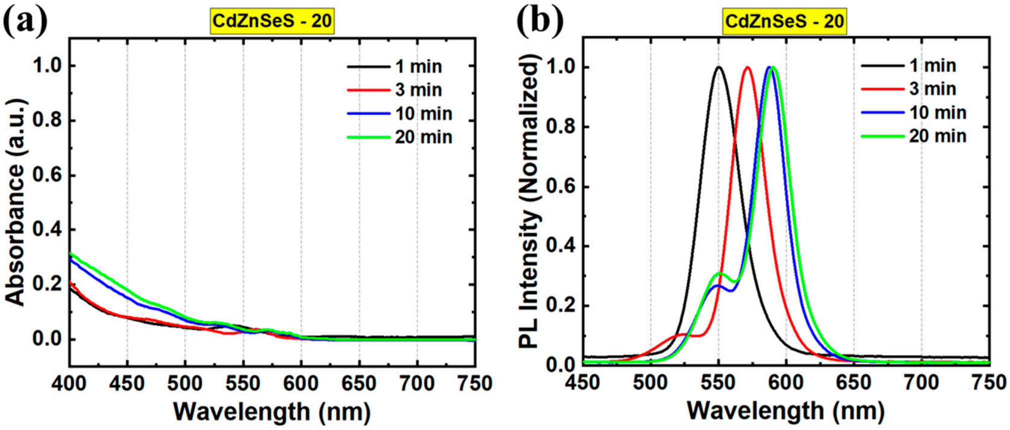

Figure S20. (**a**) Abs and (**b**) PL Spectra of Experiment A20 samples.

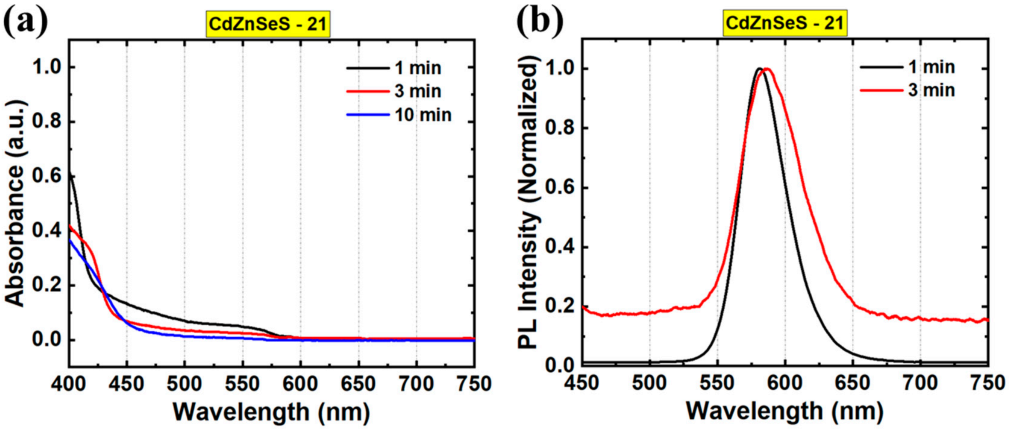

Figure S21. (**a**) Abs and (**b**) PL Spectra of Experiment A21 samples.

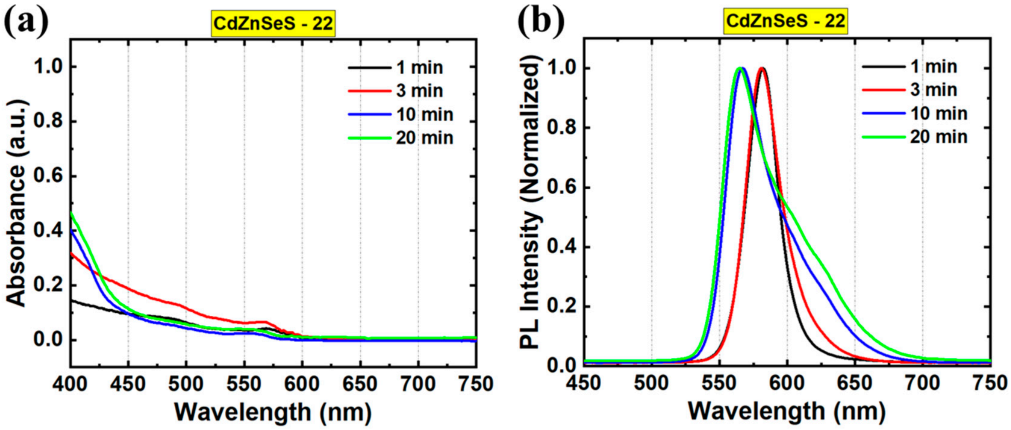

Figure S22. (**a**) Abs and (**b**) PL Spectra of Experiment A22 samples.

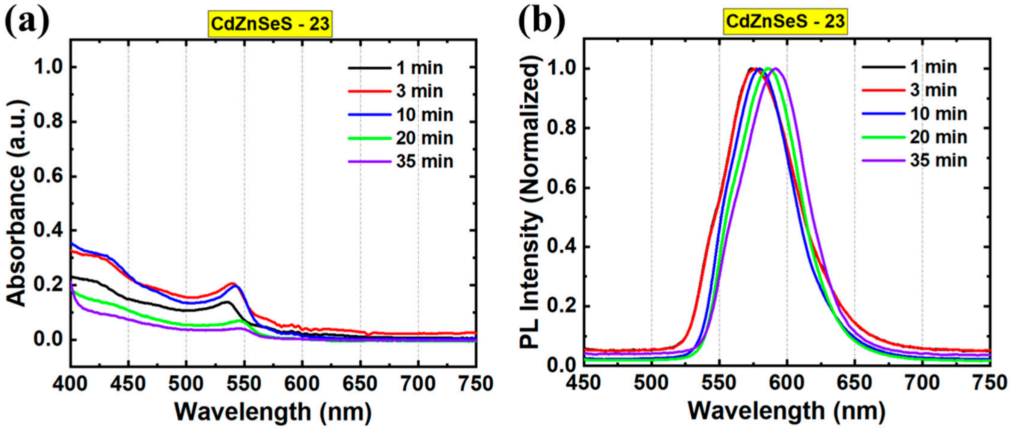

Figure S23. (**a**) Abs and (**b**) PL Spectra of Experiment A23 samples.

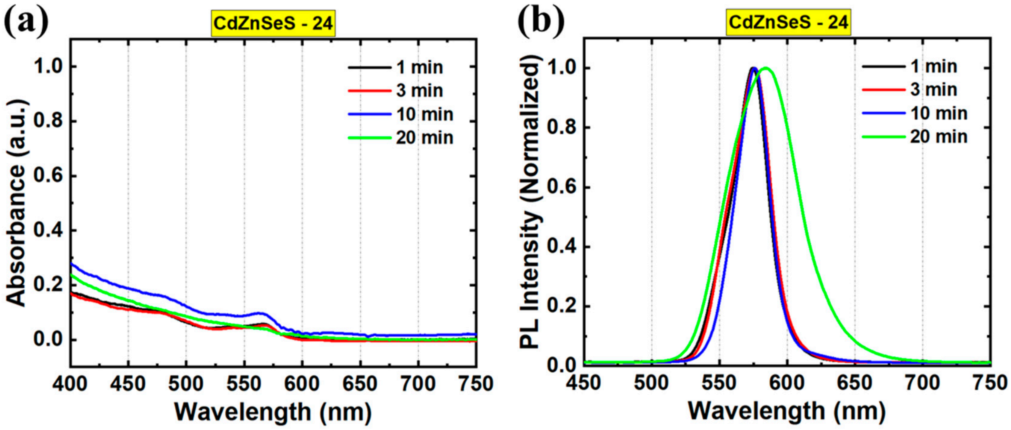

Figure S24. (**a**) Abs and (**b**) PL Spectra of Experiment A24 samples.

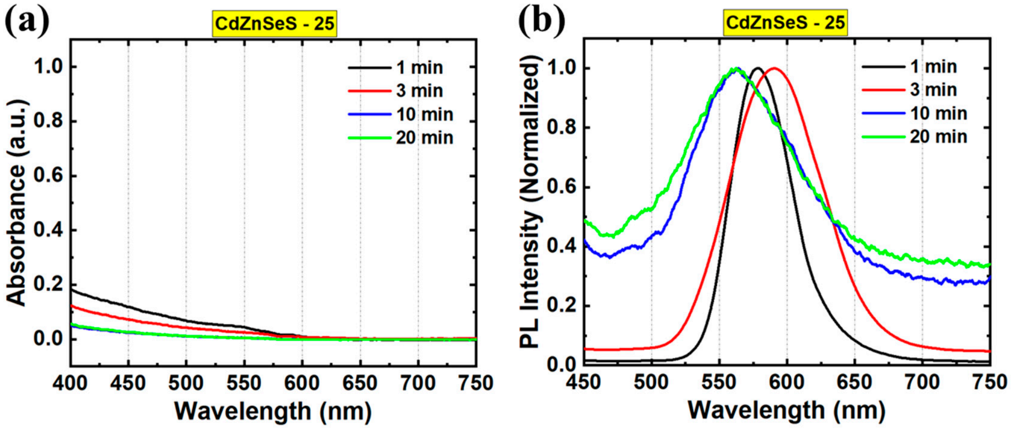

Figure S25. (**a**) Abs and (**b**) PL Spectra of Experiment A25 samples.

(a)

Level	Cd/Zn Ratio	Se/S Ratio	(Cd+Zn)/(Se+S) Ratio	OAc/(Cd+Zn) Ratio	Temperature (℃)
1	20.0	9.2	22.0	12.6	22.0
2	19.8	18.0	14.6	14.6	12.6
3	18.0	30.0	16.6	14.8	20.0
4	16.0	14.6	18.2	14.6	14.8
5	8.8	10.8	11.2	26.0	13.2
Delta	11.2	20.8	10.8	13.4	9.4
Ranking	3	1	4	2	5

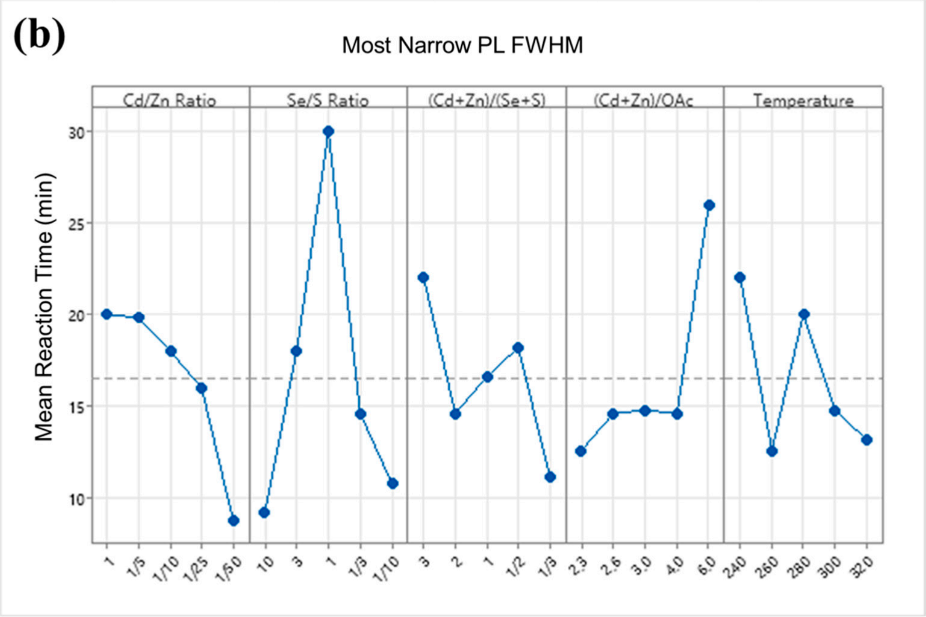

Figure S26. (**a**) Mean response table and (**b**) main effects plot for mean reaction time of samples having most narrow PL FWHM.

(a)

Level	Cd/Zn Ratio	Se/S Ratio	(Cd+Zn)/(Se+S) Ratio	OAc/(Cd+Zn) Ratio	Temperature (℃)
1	617.8	597.4	590.5	588.0	595.4
2	591.5	591.8	576.3	599.5	597.4
3	587.1	590.9	585.4	597.6	589.2
4	573.5	577.8	595.1	579.6	587.4
5	578.9	590.8	601.6	584.2	579.4
Delta	44.4	19.6	25.3	19.9	18.0
Ranking	1	4	2	3	5

(b)

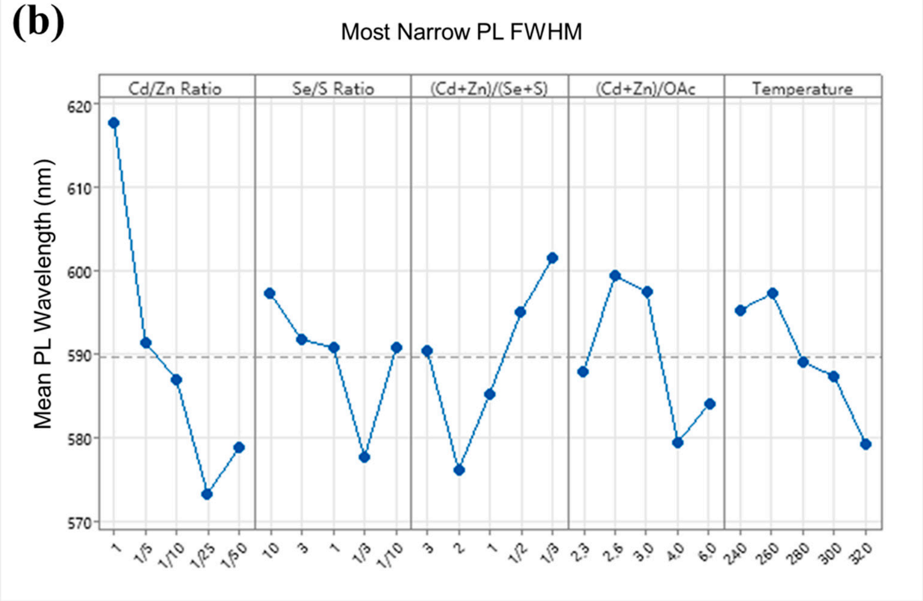

Figure S27. (**a**) Mean response table and (**b**) main effects plot for mean PL wavelength of samples having most narrow PL FWHM.

(a)

Level	Cd/Zn Ratio	Se/S Ratio	(Cd+Zn)/(Se+S) Ratio	OAc/(Cd+Zn) Ratio	Temperature (℃)
1	39.94	45.02	31.22	47.88	42.12
2	41.66	46.08	39.50	45.28	40.48
3	43.58	49.60	41.74	42.52	51.04
4	41.50	34.04	49.22	40.10	39.82
5	49.04	40.98	54.04	39.94	42.26
Delta	9.10	15.56	22.82	7.94	11.22
Ranking	4	2	1	5	3

(b)

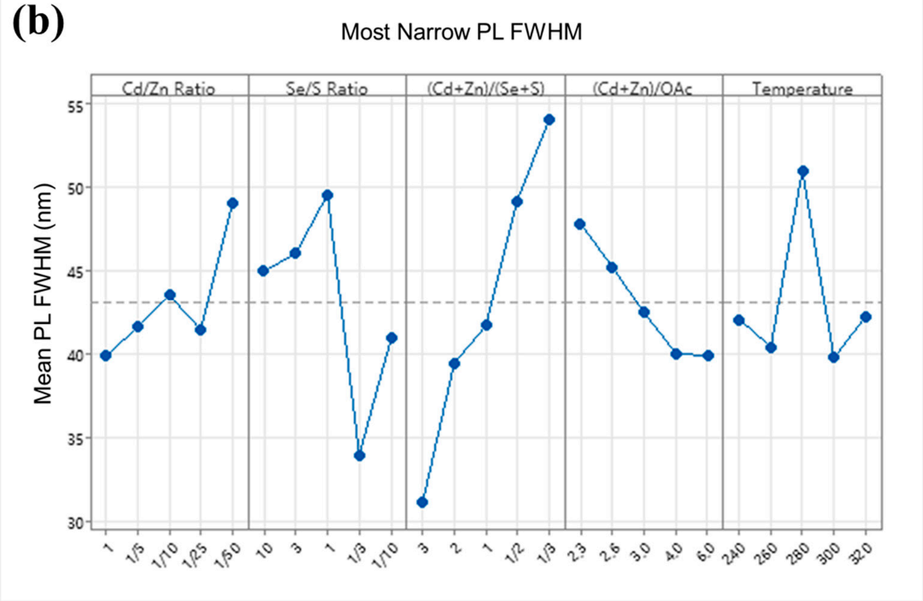

Figure S28. (**a**) Mean response table and (**b**) main effects plot for mean PL FWHM of samples having most narrow PL FWHM.

(a)

Level	Cd/Zn Ratio	Se/S Ratio	(Cd+Zn)/(Se+S) Ratio	OAc/(Cd+Zn) Ratio	Temperature (℃)
1	7.64	15.52	41.54	26.30	9.44
2	17.32	16.28	29.82	26.56	18.26
3	28.16	25.22	22.34	11.48	20.94
4	35.92	22.44	16.16	21.24	37.02
5	24.34	33.92	3.52	27.80	27.72
Delta	28.20	18.40	38.02	16.32	27.58
Ranking	2	4	1	5	3

(b)

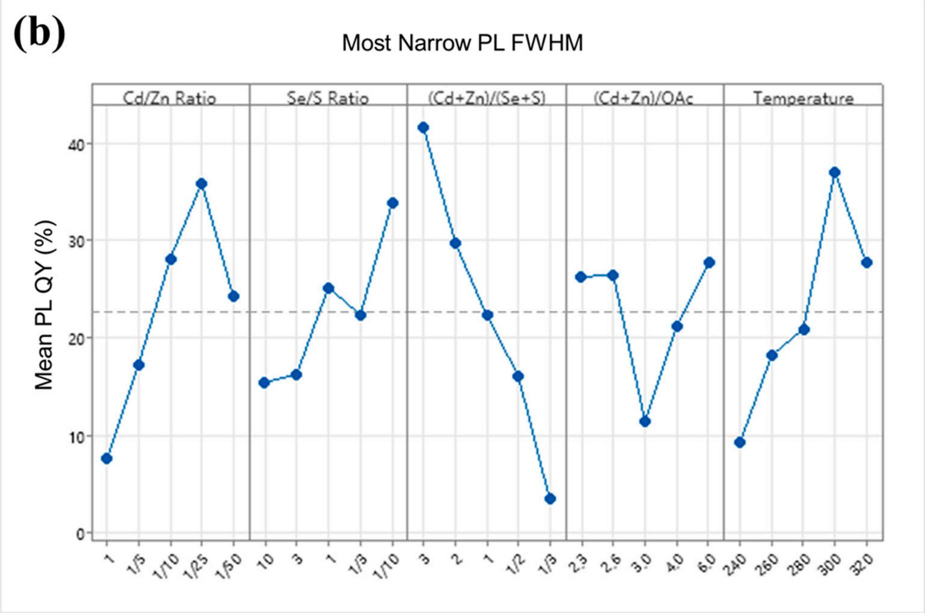

Figure S29. (**a**) Mean response table and (**b**) main effects plot for mean PL QY of samples having most narrow PL FWHM.

(a)

Level	Cd/Zn Ratio	Se/S Ratio	(Cd+Zn)/(Se+S) Ratio	OAc/(Cd+Zn) Ratio	Temperature (℃)
1	17.8	14.2	21.6	12.6	24.2
2	17.6	19.8	24.2	15.6	16.6
3	19.6	20.6	19.2	14.4	8.6
4	18.8	19.2	10.8	29.2	29.2
5	7.0	7.0	5.0	9.0	2.2
Delta	12.6	13.6	19.2	20.2	27.0
Ranking	5	4	3	2	1

(b)

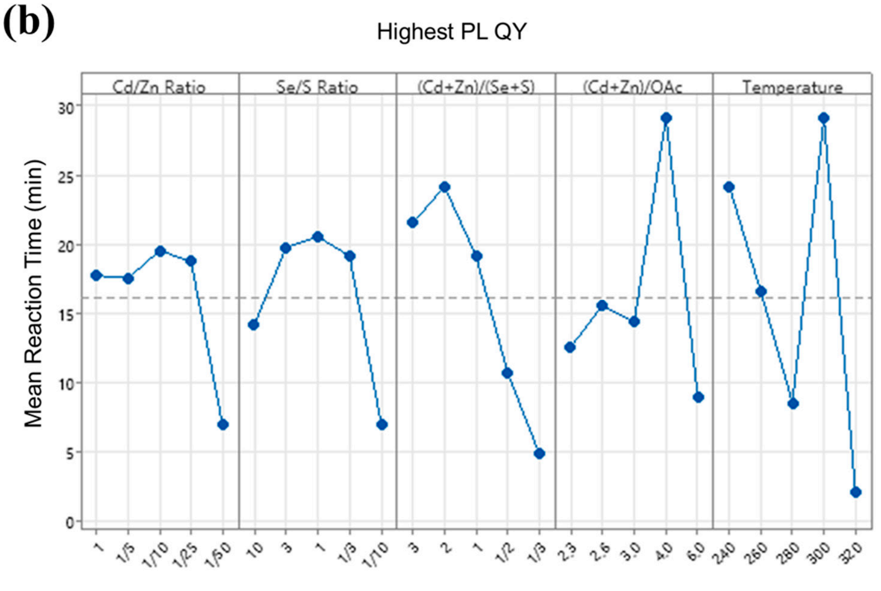

Figure S30. (**a**) Mean response table and (**b**) main effects plot for mean reaction time of samples having highest PL QY.

(a)

Level	Cd/Zn Ratio	Se/S Ratio	(Cd+Zn)/(Se+S) Ratio	OAc/(Cd+Zn) Ratio	Temperature (℃)
1	609.8	596.2	589.1	589.7	588.8
2	591.7	596.6	580.0	600.2	600.8
3	590.1	589.2	590.3	595.8	588.7
4	577.2	584.1	597.9	579.5	588.4
5	580.4	583.1	591.9	584.1	582.4
Delta	32.6	13.5	17.9	20.7	18.4
Ranking	1	5	4	2	3

(b)

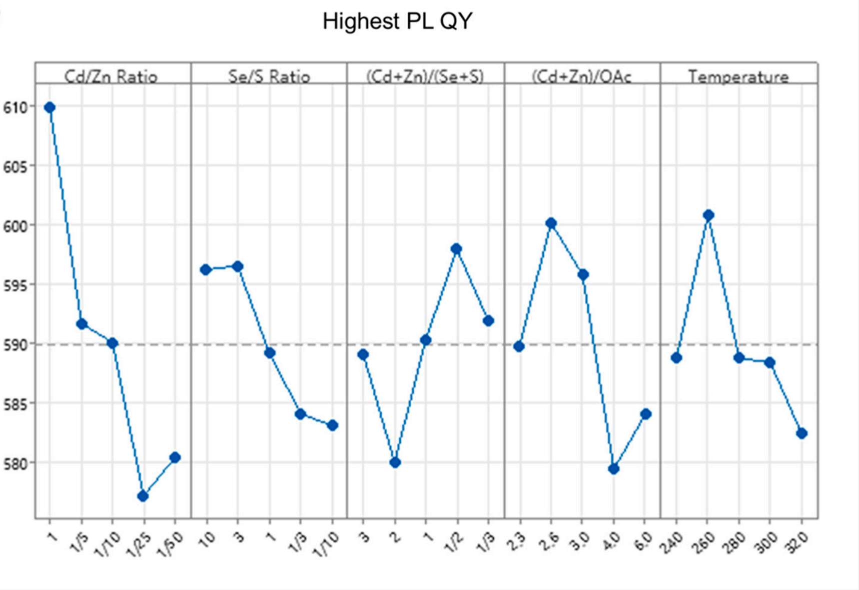

Figure S31. (**a**) Mean response table and (**b**) main effects plot for mean PL wavelength of samples having highest PL QY.

(a)

Level	Cd/Zn Ratio	Se/S Ratio	(Cd+Zn)/(Se+S) Ratio	OAc/(Cd+Zn) Ratio	Temperature (℃)
**1**	50.18	45.52	33.54	52.62	49.50
**2**	52.62	44.44	37.30	45.96	43.20
**3**	44.46	53.44	46.04	47.70	53.16
**4**	41.96	40.68	53.00	38.88	46.02
**5**	42.56	47.70	61.90	46.62	39.88
**Delta**	10.66	12.76	28.36	13.74	13.28
**Ranking**	5	4	1	2	3

(b)

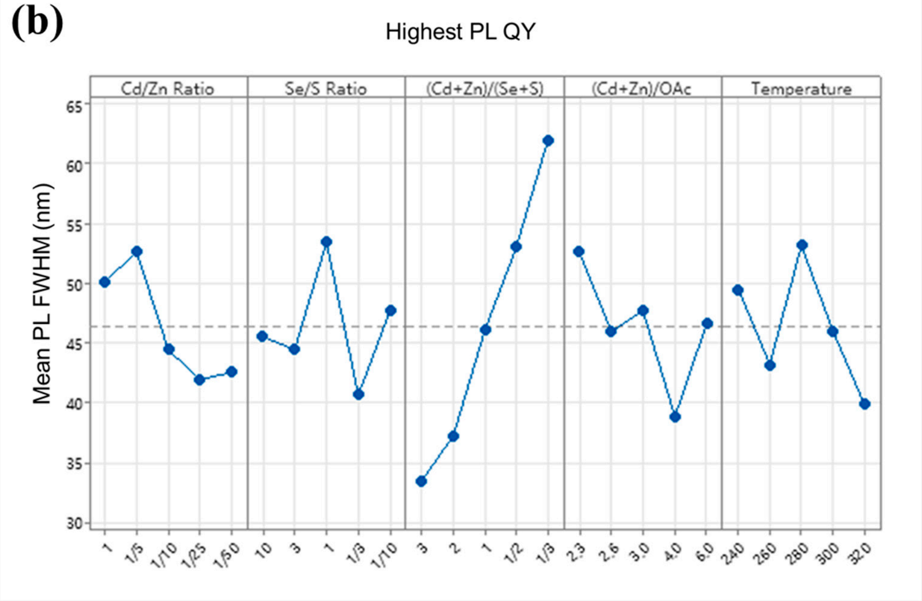

Figure S32. (**a**) Mean response table and (**b**) main effects plot for mean PL FWHM of samples having highest PL QY.

(a)

Level	Cd/Zn Ratio	Se/S Ratio	(Cd+Zn)/(Se+S) Ratio	OAc/(Cd+Zn) Ratio	Temperature (℃)
1	9.78	22.40	43.80	30.22	10.10
2	25.30	22.40	35.02	27.08	27.68
3	29.46	27.78	28.10	17.42	21.42
4	38.12	28.00	17.30	25.04	40.94
5	31.90	33.98	10.34	34.80	31.42
Delta	28.34	11.58	33.46	17.38	30.84
Ranking	3	5	1	4	2

(b)

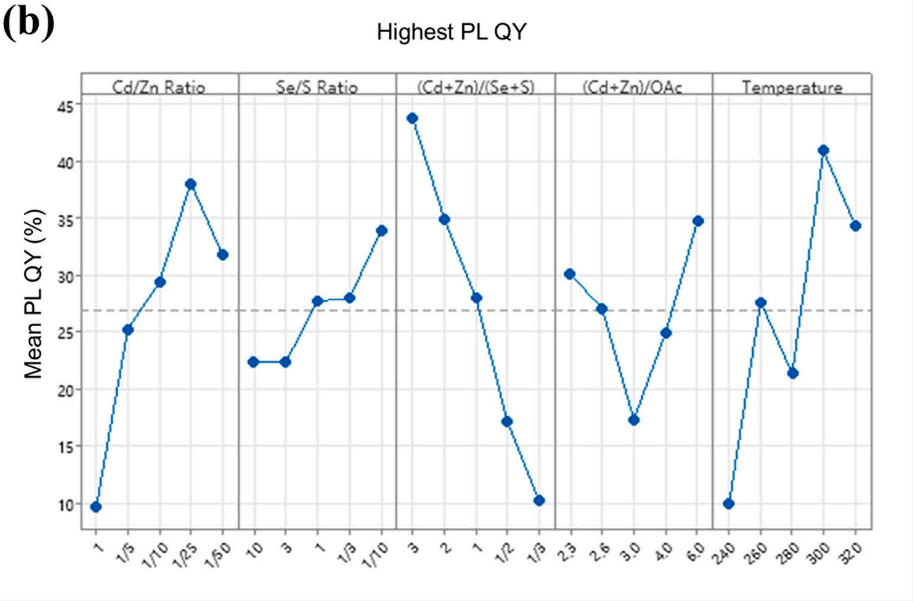

Figure S33. (**a**) Mean response table and (**b**) main effects plot for mean PL QY of samples having highest PL QY.

(a)

Level	Temperature (℃)	Zn Source	S Source
1	540.9	541.6	541.1
2	541.9	541.5	540.3
3	540.8	540.5	542.3
Delta	1.0	1.1	2.1
Ranking	3	2	1

(b)

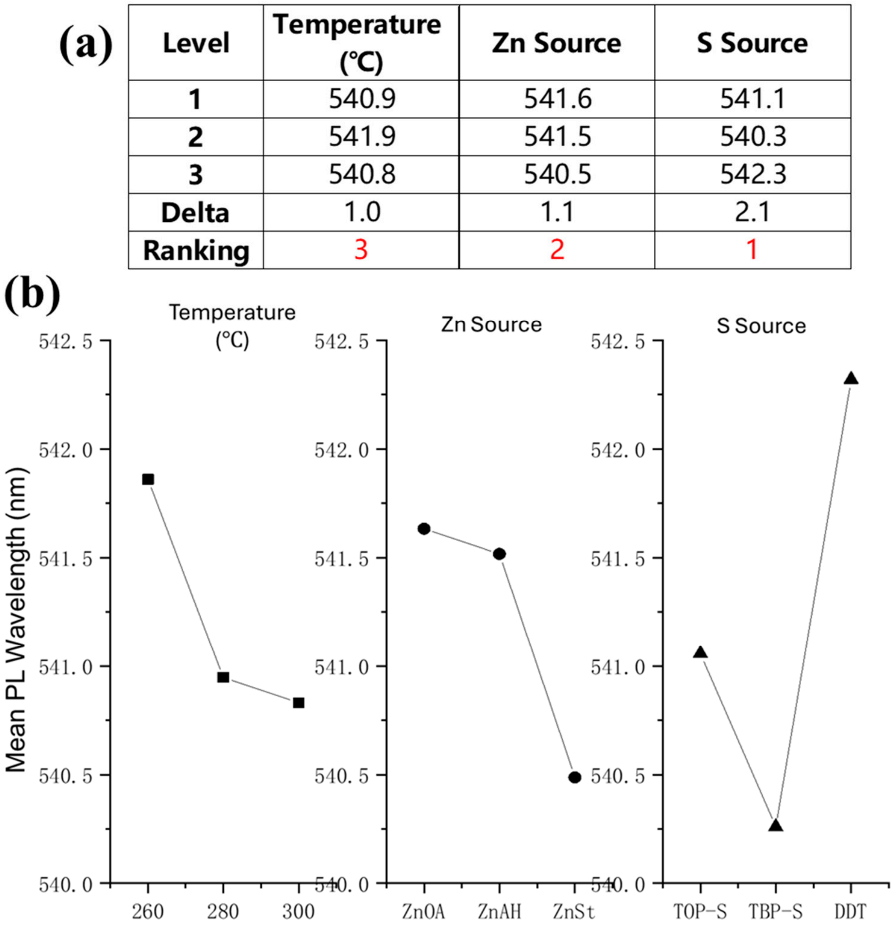

Figure S34. (**a**) Mean response table and (**b**) main effects plot for mean PL wavelength of samples.

(a)

Level	Temperature (℃)	Zn Source	S Source
1	23.75	23.89	23.72
2	23.63	23.60	23.50
3	23.58	23.47	23.74
Delta	0.17	0.43	0.25
Ranking	3	1	2

(b)

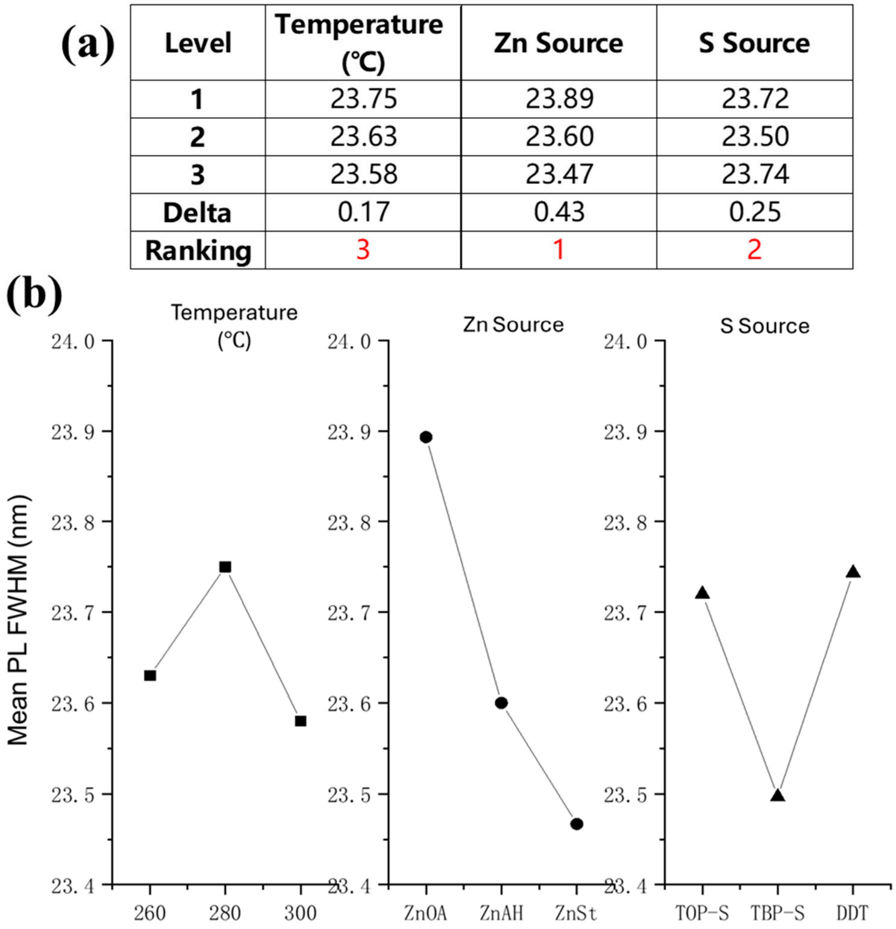

Figure S35. (**a**) Mean response table and (**b**) main effects plot for mean PL FWHM of samples.

(a)

Level	Temperature (℃)	Zn Source	S Source
1	83.73	85.17	79.47
2	82.63	81.63	84.17
3	84.97	84.53	87.70
Delta	2.33	3.53	8.23
Ranking	3	2	1

(b)

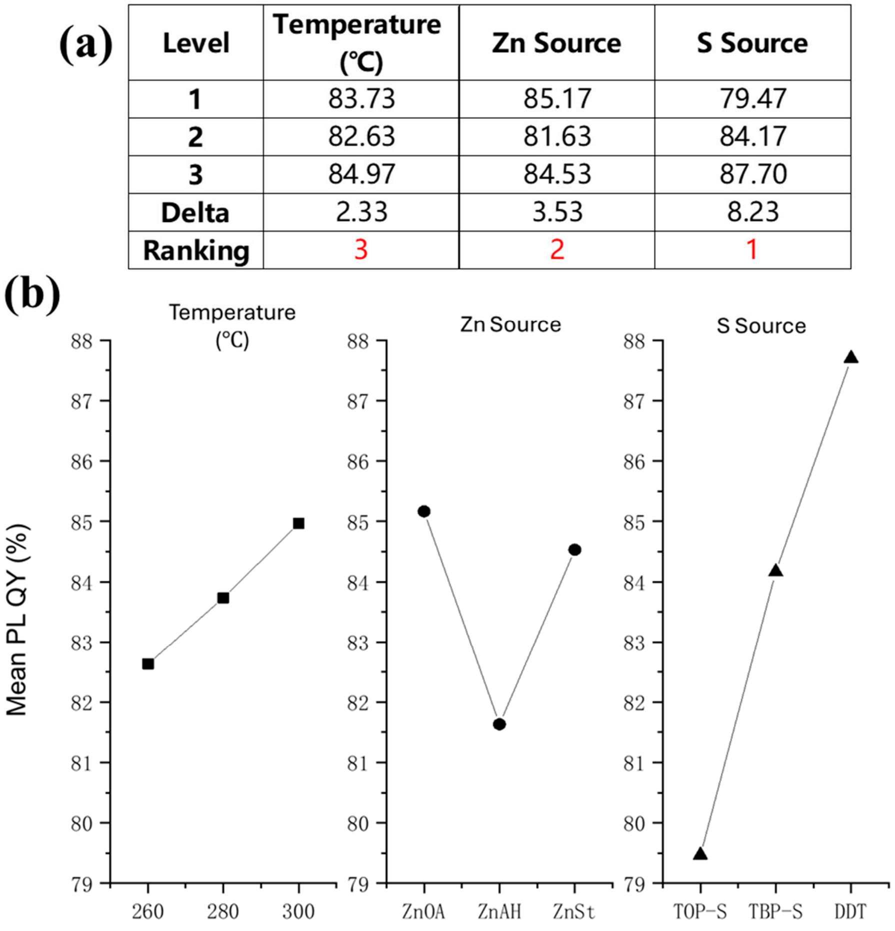

Figure S36. (**a**) Mean response table and (**b**) main effects plot for mean PL QY of samples.

(a)

Level	Temperature (℃)	Zn Source	S Source
1	70.05	67.64	76.32
2	60.15	69.91	64.23
3	69.98	62.62	59.63
Delta	9.90	7.29	16.69
Ranking	2	3	1

(b)

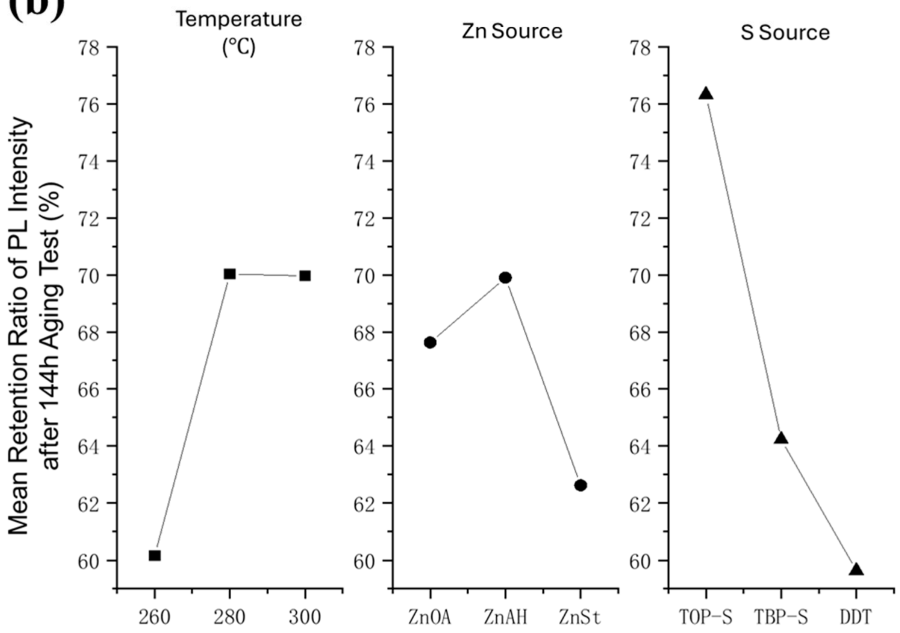

Figure S37. (**a**) Mean response table and (**b**) main effects plot for mean retention ratio of PL intensity after 144h aging test of samples.

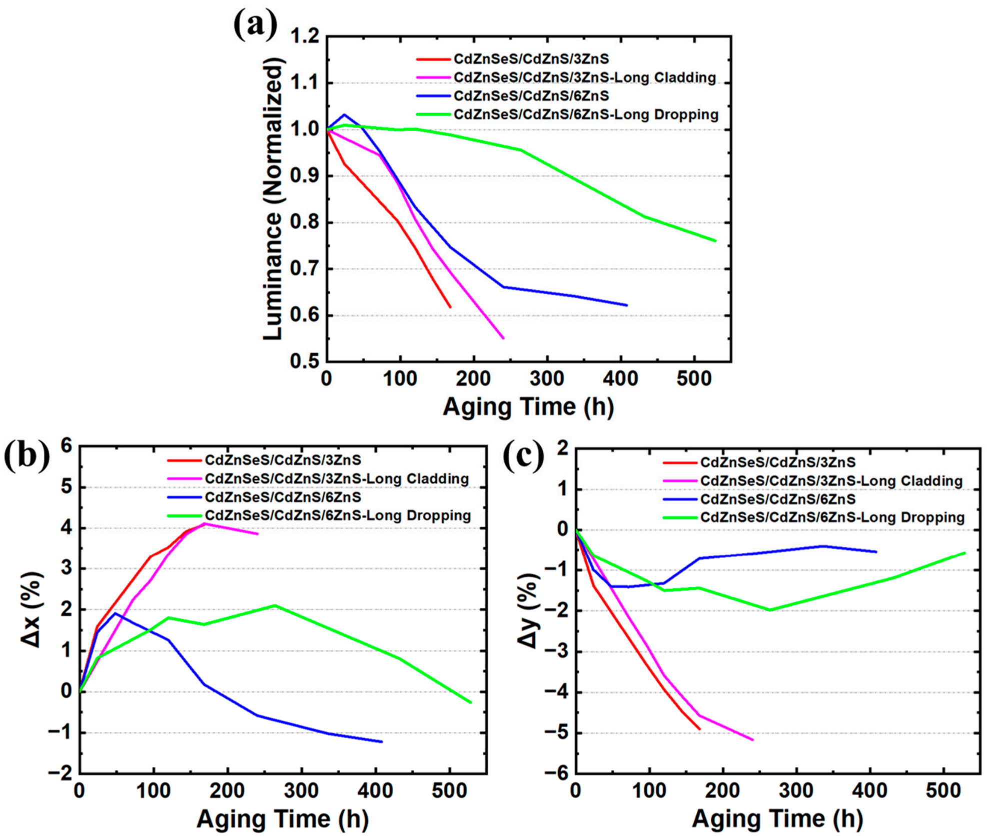

Figure S38. Monitoring and change results of (**a**) brightness, (**b**) x chromaticity coordinate, (**c**) y chromaticity coordinate of quantum dot masterbatches under blue light + double-85 aging test.

Evidence for ligand exchange can be provided by Fourier transform infrared spectroscopy (FTIR) measurements. As shown in Figure S39 below, the black and red lines represent the FTIR transmission spectra of quantum dots before and after ligand exchange, respectively.

The light blue shaded area marks three representative characteristic peaks that disappeared after ligand exchange, indicating that most oleic acid (OAc) ligands were replaced: (1) The peak at 3005 cm^{-1} corresponds to the stretching vibration of C-H in carbon-carbon double bonds (C=C-H), which vanished after ligand exchange. (2) The peak at 1713 cm^{-1} corresponds to the stretching vibration of C=O, and the carboxyl peak signal of oleic acid disappeared after ligand exchange. (3) The peaks in the range of 1500–1600 cm^{-1} correspond to the skeletal vibration of C=C double bonds, which also disappeared after ligand exchange.

The green arrows indicate two new representative characteristic peaks that emerged after ligand exchange, confirming the successful grafting of dodecylphosphonic acid (TDPA) ligands: (1) A new peak appeared at 1240 cm^{-1}, corresponding to the stretching vibration of the phosphate group (P=O), indicating the introduction of TDPA. (2) A new peak appeared at 1070 cm^{-1}, corresponding to the stretching vibration of P-O bonds, further confirming the presence of TDPA.

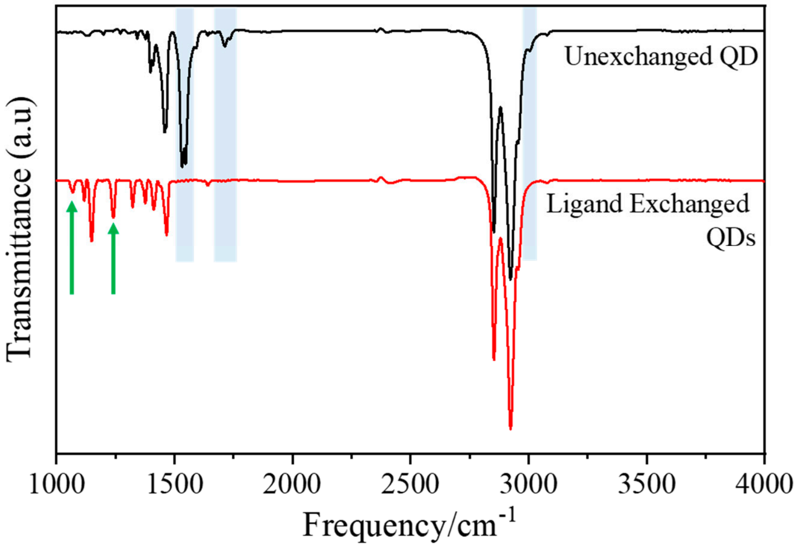

Figure S39. Fourier transform infrared spectroscopy (FTIR) transmission spectra of QDs before (black line) and after (red line) ligand exchange.

The emission and excitation spectra of pure QDs and QDs embedded in PS are shown in Figure S40 below. Comparing the emission spectra in Figure S40(**a**) and S40(**b**), and the excitation spectra in Figure S40(**c**) and S40(**d**), there is almost no change. This indicates that the PS matrix can well preserve the intrinsic luminescent properties of QDs without affecting their excitation and emission.

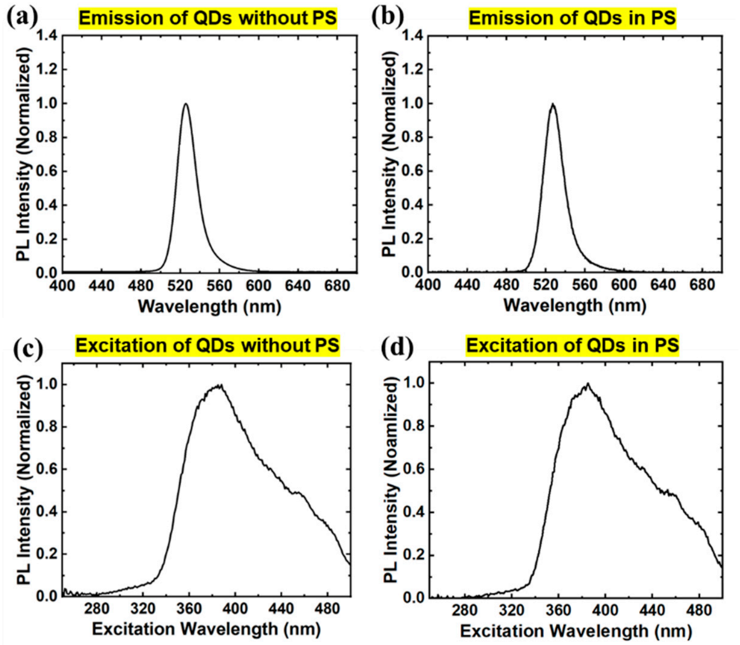

Figure S40. (**a**) Emission spectrum of pure QDs; (**b**) Emission spectrum of QDs in PS; (**c**) Excitation spectrum of pure QDs; (**d**) Excitation spectrum of QDs in PS.
